# Differential tissue specific, temporal and spatial expression patterns of the Aggrecan gene is modulated by independent enhancer elements

**DOI:** 10.1038/s41598-018-19186-4

**Published:** 2018-01-17

**Authors:** Ian M. H. Li, Ke Liu, Alice Neal, Peter D. Clegg, Sarah De Val, George Bou-Gharios

**Affiliations:** 1 Institute of Ageing and Chronic Disease, University of Liverpool, William Henry Duncan Building, Liverpool, L7 8TX UK; 2 Ludwig Cancer Research Ltd, University of Oxford, Oxford, UK

## Abstract

The transcriptional mechanism through which chondrocytes control the spatial and temporal composition of the cartilage tissue has remained largely elusive. The central aim of this study was to identify whether transcriptional enhancers played a role in the organisation of the chondrocytes in cartilaginous tissue. We focused on the Aggrecan gene (*Acan*) as it is essential for the normal structure and function of cartilage and it is expressed developmentally in different stages of chondrocyte maturation. Using transgenic reporter studies in mice we identified four elements, two of which showed individual chondrocyte developmental stage specificity. In particular, one enhancer (−80) distinguishes itself from the others by being predominantly active in adult cartilage. Furthermore, the −62 element uniquely drove reporter activity in early chondrocytes. The remaining chondrocyte specific enhancers, +28 and −30, showed no preference to chondrocyte type. The transcription factor SOX9 interacted with all the enhancers *in vitro* and mutation of SOX9 binding sites in one of the enhancers (−30) resulted in a loss of its chondrocyte specificity and ectopic enhancer reporter activity. Thus, the *Acan* enhancers orchestrate the precise spatiotemporal expression of this gene in cartilage types at different stages of development and adulthood.

## Introduction

The mammalian genome has an abundance of regulatory elements compared to coding sequences. As cells differentiate and commit to a lineage fate the environment surrounding the DNA changes. The regulatory elements, histone modifications, transcription factors (TFs) and 3D topography delineate and fine-tune the activation and repression of the genes that defines the cell^1,2^. In many genes that are expressed in multiple cell types, systematic mapping of chromatin state and 3D chromosome conformation across cell types revealed separate regulatory elements that direct expression in a spatial and temporal manner^3–6^. One of the characteristics of these regulatory elements, also known as enhancers, is that they arrange and co-operate in clusters. Additionally, there is a high degree of functional redundancy of these enhancers in reporter assays and in transcription factor occupancy^7,8^. The role of these regions and why the genome contains so many are currently being examined and their importance in normal homeostasis and disease are becoming increasingly apparent^9,10^.

The Aggrecan gene (*Acan*) codes for a large aggregating chondroitin sulphate proteoglycan, the main structural proteoglycan in cartilage; found in the growth plates and articular cartilage lining synovial joints. Acan does not exist in isolation within the extracellular matrix of cartilage. Instead, it is present in the form of proteoglycan aggregates^11^. Up to 100 individual monomers of Acan are non-covalently attached to a central filament of hyaluronic acid forming macromolecular aggregates of Acan^12^. Even though high levels of Acan expression are found predominantly in cartilage, its mRNA has also been observed in other tissues including central nervous system^13^, and in the notochord giving rise to the nucleus pulposus of the intervertebral discs (IVD)^14^.

Loss of Acan during mouse development results in post-natal lethality as it is essential for the formation of the body’s skeletal elements^15^. Furthermore, the prevalence of conditions, such as osteoarthritis (OA), the most common disease of the musculoskeletal system that occurs predominantly in older adults, is exhibited from an early stage by a significant downregulation of *Acan* expression^16,17^. Additionally, shorter fragments of *Acan* are associated with osteoarthritic joint disease and ageing^18^. This represents a characteristic and functionally important process paralleling cartilage degeneration by metalloproteinases (MMPs) and aggrecanases (ADAMTS) that result in OA progression. Therefore, a complete understanding of the transcriptional network that regulates *Acan* during chondrogenesis and the balance of transcription of the protein in adulthood may allow us to modulate its expression in pathological conditions and enable us to understand how a heterogeneous population of chondrocytes produce specific tissues such as articular cartilage and the growth plate^19^.

Previous studies identified a chondrocyte-specific enhancer located at −10 kb upstream of the *Acan* transcription start site (TSS), termed A1^20^. The activity of this enhancer was confined to differentiated chondrocytes and was regulated by the transcription factor SOX9 with the aid of L-SOX5 and SOX6^20^. Additionally, a zebrafish study that screened human *Acan cis*-regulatory elements based on sequence conservation identified 11 elements capable of driving GFP-reporter activity in larvae^21^. Furthermore, cell-based screens for candidate enhancers hinted at the existence of a potent enhancer 30 kb upstream of the *Acan* TSS. More recently ChIP-seq experiments using SOX9 have established clusters or super enhancers that span up to 140 kb down-stream of the *Acan* TSS^22–25^. These studies provided two key insights into the *Acan* regulation; the first is that these enhancers are functional in differentiated chondrocytes. This is particularly true in articular cartilage and growth plates. However, these enhancers required insertion of multiple copies in tandem. Secondly, *Acan* appears to be regulated by multiple enhancers or super-enhancers, which have not been functionally validated but were identified by SOX9 binding and histone marks. Therefore, we sought to establish how chondrocytes establish differential cartilage tissue composition in different areas of the developing and developed body with *Acan* as our focus. We identify four enhancers that exhibit overlapping spatial and temporal activities such that, together, they likely contribute to ensure the robust expression of *Acan* in all cartilage types throughout development and in adult tissue.

## Results

### Identification of differential activities for four Acan enhancers in developing cartilage

**Figure 1:**
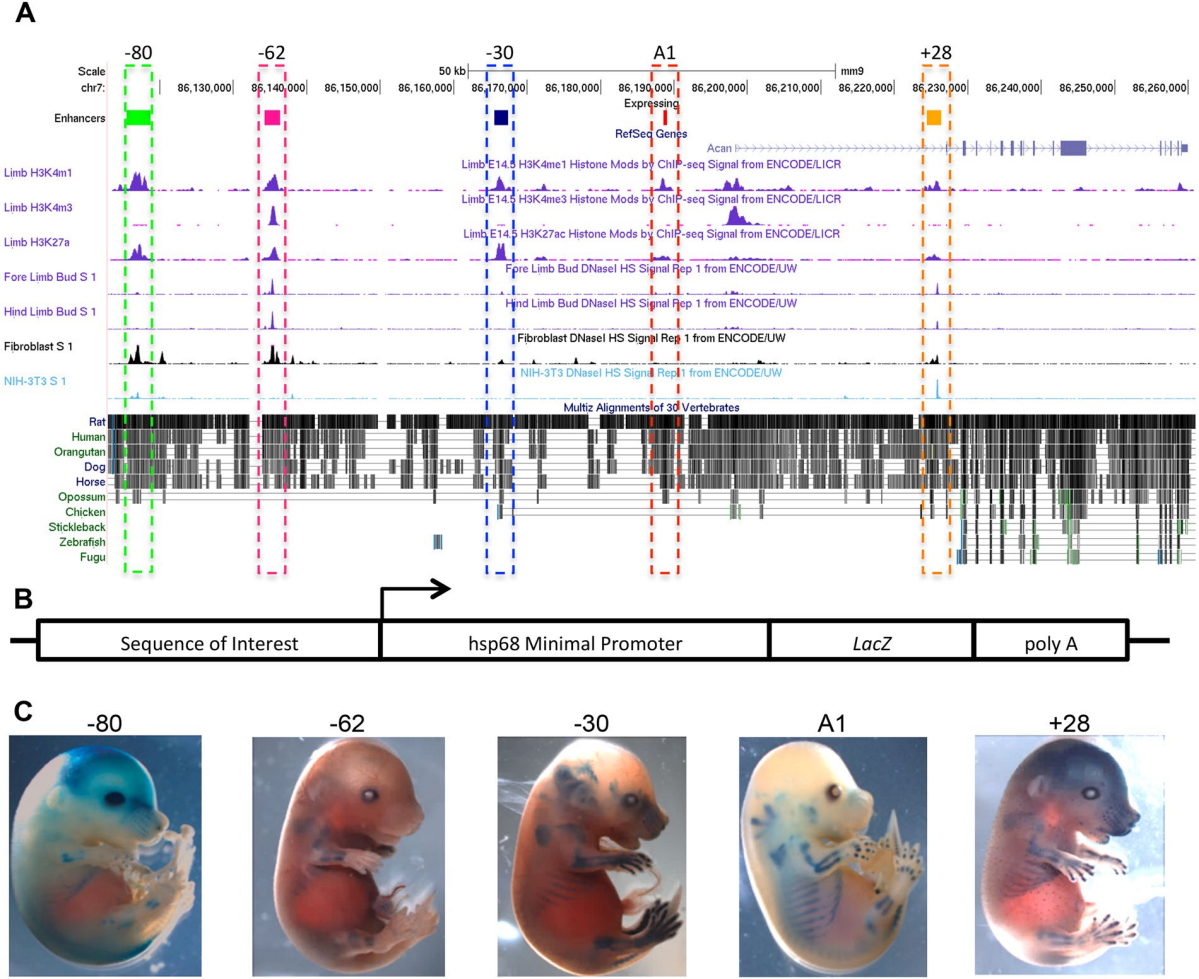
Identification of *Aggrecan* enhancers. (**A**) ENCODE analysis of the mouse *Acan* locus. Peaks show strong indication of enhancers using H3K4me1 and H3K27ac along with conservation. The selected regions for analysis are highlighted, +28, −10(A1), −30, −62 and −80. (**B**) Schematic of the HSP68-*LacZ*-GW reporter vector that was used to create transgenic mice for analysis. (**C**) Representative whole mount images of transgenic embryos at E15.5 for each enhancer region tested showing a variation of transgene activity

We used the availability of data from the Encyclopaedia of DNA Elements (ENCODE) project for mouse embryo limb at embryonic day 14.5 (E14.5) enhancer marks, such as the H3K4me1/2 (poised and active enhancers) and H3K27Ac (active enhancers) as well as phylogenic conservation to identify multiple *cis-*regulatory elements within and around the *Acan* gene. During the data collection of this study SOX9 (the master regulator of chondrocyte fate) and histone modifications ChIP-seq data from three groups, one in rats^24^ and the other two in new born mouse rib chondrocytes and limb bud (E12.5) became available^23,25^, this allowed us to validate that the regions we had selected had chondrocyte specific enhancer markers (Supplementary Figure S1). In addition to the already reported −10 (A1) *Acan* enhancer^20^, we identified four regions, one in the first intron at +28 kb (refer to as +28) relative to the *Acan* TSS and 3 upstream regions at −30, −62 and −80 kb (referred to as −30, −62 and −80 in the rest of the manuscript) that showed strong indications for enhancer activity (Fig. 1A).

The intergenic regions at −30 and −80 both contained histone marks considered indicative of enhancers, H3K4me1 and H3K27ac, and were void of H3K4me3 marks in E14.5 limbs, but lacked enhancer histone signatures in E11.5 limb buds, suggestive of possible enhancers in these regions at later developmental stages, as shown in Fig. 1A. The intronic element, +28, found in the first intron of *Acan* lacked H3K4me3 but contained noticeable signal for limb specific histone modification for enhancers and DNaseI hypersensitivity at E11.5 and E14.5. Similar DNaseI hypersensitivity was noted in fibroblast cells in both −62 and −80 regions.

To experimentally verify and characterise the activity of these enhancer regions, each sequence was cloned upstream of the silent HSP68-LacZ-GW^26^ vector containing a ß-galactosidase (β-gal) reporter (Fig. 1B). Transgenic mouse embryos were made and were analysed for transgene activity by β-gal staining at E15.5. Each enhancer was found to be active in at least a subset of cartilage elements (Fig. 1C). We therefore generated mouse transgenic mouse lines to analyse the spatial and temporal patterns of activity of each enhancer.

**Figure 2:**
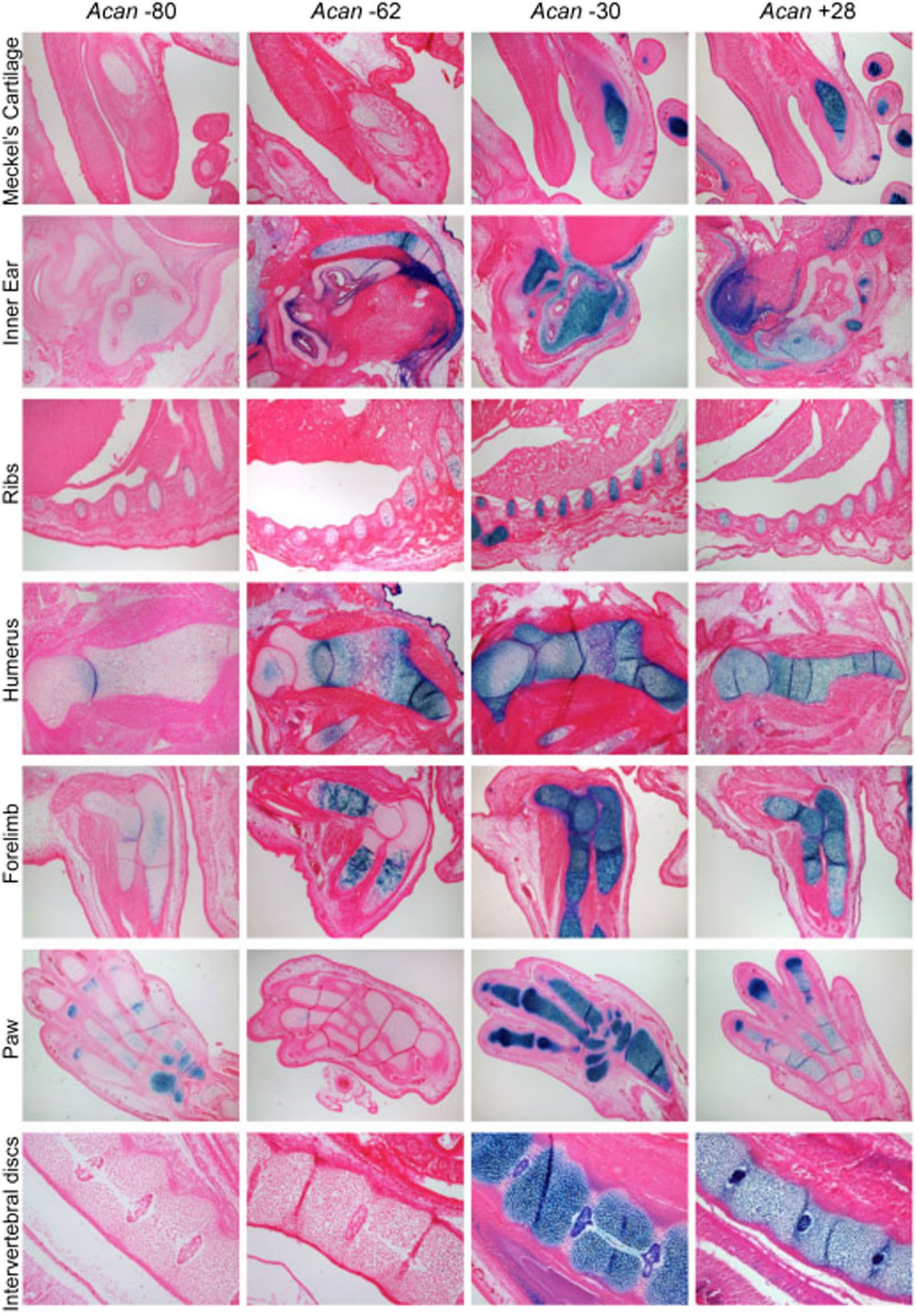
Histological examination of transgenic mice at E15.5. β-gal staining with a counter-stain of eosin of the Meckel’s cartilage, Inner ear, ribs, humerus, radius and ulna of the forelimb and intervertebral discs (IVD) of the lumbar spine. The +28 and −30 show similarities in chondrocytes. The −62 element shows no activity in the IVD but in the hypertrophic regions of the limb, humerus, inner ear and ribs β-gal can be detected but no staining in the distal elements like the paw and in Meckel’s cartilage. The −80 at this stage is weaker than the other enhancers; its activity is scattered and more favourable to the joints in many articulations.

Whole mount staining and sections of E15.5 embryos showed that +28 and −30 elements shared X-gal staining patterns in all cartilage elements of the limbs, ribs, primordial otic region, jaw, vertebrae, and IVDs (Supplementary Table S1, Fig. 2). Additionally, parts of the ear that undergo endochondral ossification, such as the cartilage primordial of the temporal and occipital bones of the chondrocranium, were stained. In addition to chondrocyte transgene activity, we have noted +28 enhancer activity in hair follicles which could be indicative of Acan within this tissue. Indeed, aggrecan has been shown to be present surrounding the hair follicle cells^27^.

Sections of E15.5 embryos harbouring the −62 region revealed clear differences when compared to embryos containing other enhancers (Fig. 2). *LacZ* staining was detected in both the proximal limb elements and in the ribs, specifically in the pre and hypertrophic chondrocytes but not in skeletal elements that were distal (Fig. 2). No transgene activity was noted in the resting zone of the growth plate, in the Meckel’s cartilage and the IVD (Fig. 2). The vertebrae and the tail were lightly stained, but the otic region was stained similarly to other enhancers. We concluded from these observations that the −62 enhancer element is active mainly in the growth plates from the late proliferating stage as well as in pre-hypertrophic and hypertrophic chondrocytes. It must be noted that *Acan* is down regulated and Acan mRNA is not expressed in hypertrophic chondrocytes, the staining we see here may be remnants of the transgene expression of an earlier time pointed that is stained due to the stability of the β- galactosidase protein, but enhancer activity in these cells cannot be ruled out.

The furthest upstream enhancer region identified, the −80, appeared in whole mount embryos at E15.5 to be weaker than the other enhancers in 8 founder embryos (Supplementary Table S1, Fig. 1C). Section of embryos showed inconsistent activity of the transgene in the middle of limb and vertebra cartilage elements, stronger activity in articular cartilage regions, but no activity was noted in the IVDs (Supplementary Table S1, Fig. 2).

### Characterisation of Acan enhancer activities in adult cartilage

**Figure 3:**
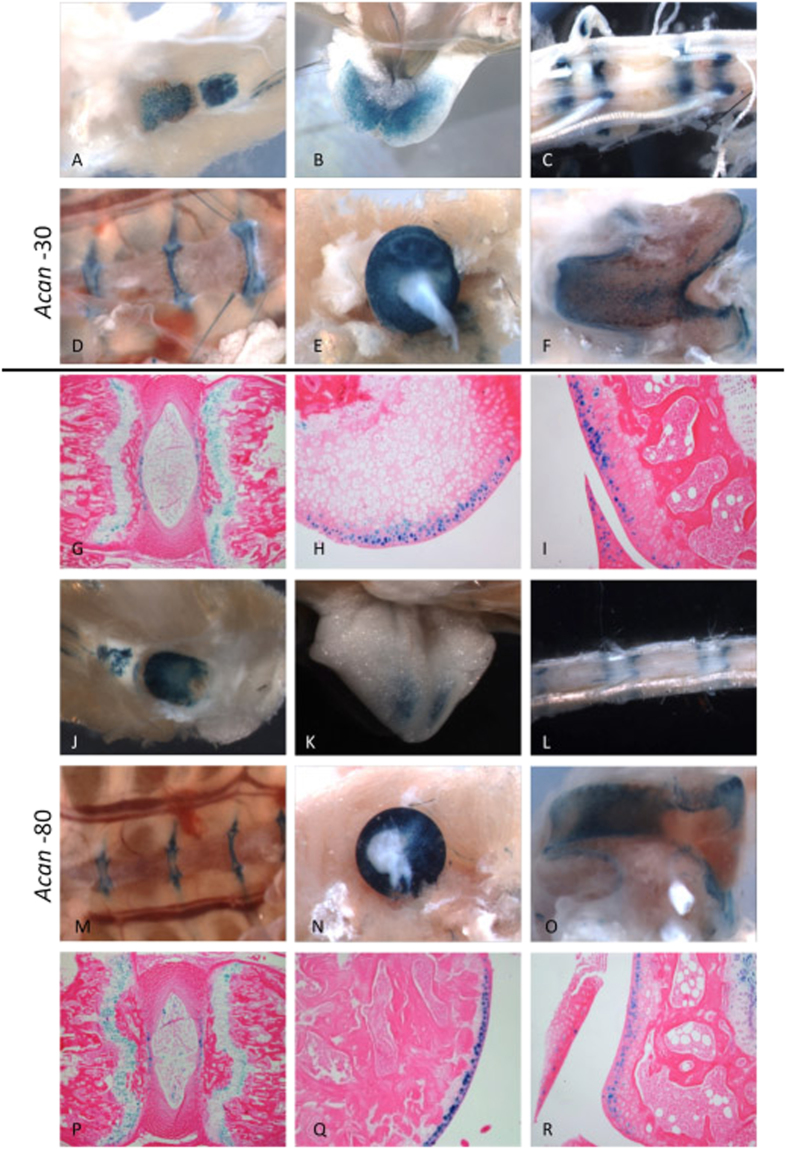
8 weeks old activity of the −30 and −80 enhancers. The −30 enhancer drives reporter expression in 8 weeks old transgenic mice, with whole mount staining showing activity in the patella (**A**), xiphoid (**B**), tail (**C**), sternum (**D**), femoral head (**E**) and condyle (**F**). There is strong X-gal staining in the cartilage end plates of the IVD, weakly in the annulus fibrosus (AF) but not present in the nucleus pulpous (NP, **G**), Activity is seen in the superficial region of the articular cartilage of the hip (**H**), and in the condyle of the knee (**I**). The −80 is strongly active at 8 weeks in whole mount. It shares similar staining patterns to the −30 in the patella (**J**), xiphoid (**K**), tail (**L**), sternum (**M**), femoral head (**N**) and condyle (**O**). Histologically there is staining in the growth plate of vertebrae and end plate of IVD (**P**). Some cells in the NP but not in the AF are stained. The superficial region of the articular cartilage in the femoral head (**Q**), condyle (**R**) are stained similar to the −30 enhancer.

We then asked whether these enhancers are also active in adult tissues. Whole-mount staining of eight-week-old mice showed −30 activity in limb articular cartilage and growth plate, and in xiphoid and sternum cartilage (Fig. 3A–F). Histological sections showed that −30 drove reporter activity in both articular and growth plate chondrocytes (Fig. 3G–I).

Interestingly, while the −80 enhancer drives limited activity in embryos, it demonstrated strong activity at 8 weeks of age in articular cartilage and growth plate chondrocytes of the limb and in the cartilage end plate of the vertebra. Other tissues in which transgene activity was observed included the xiphoid, patella and sternum (Fig. 3J–R). Additionally, minor staining was seen in the nucleus pulposus (NP) but not in the annulus fibrosus (AF) of IVDs (Fig. 3P). Similarly, staining was absent in the AF of the −30 enhancer (Fig. 3G).

### All *Acan* enhancers contain SOX9-like binding elements and interact with SOX9 *in vitro*

**Figure 4:**
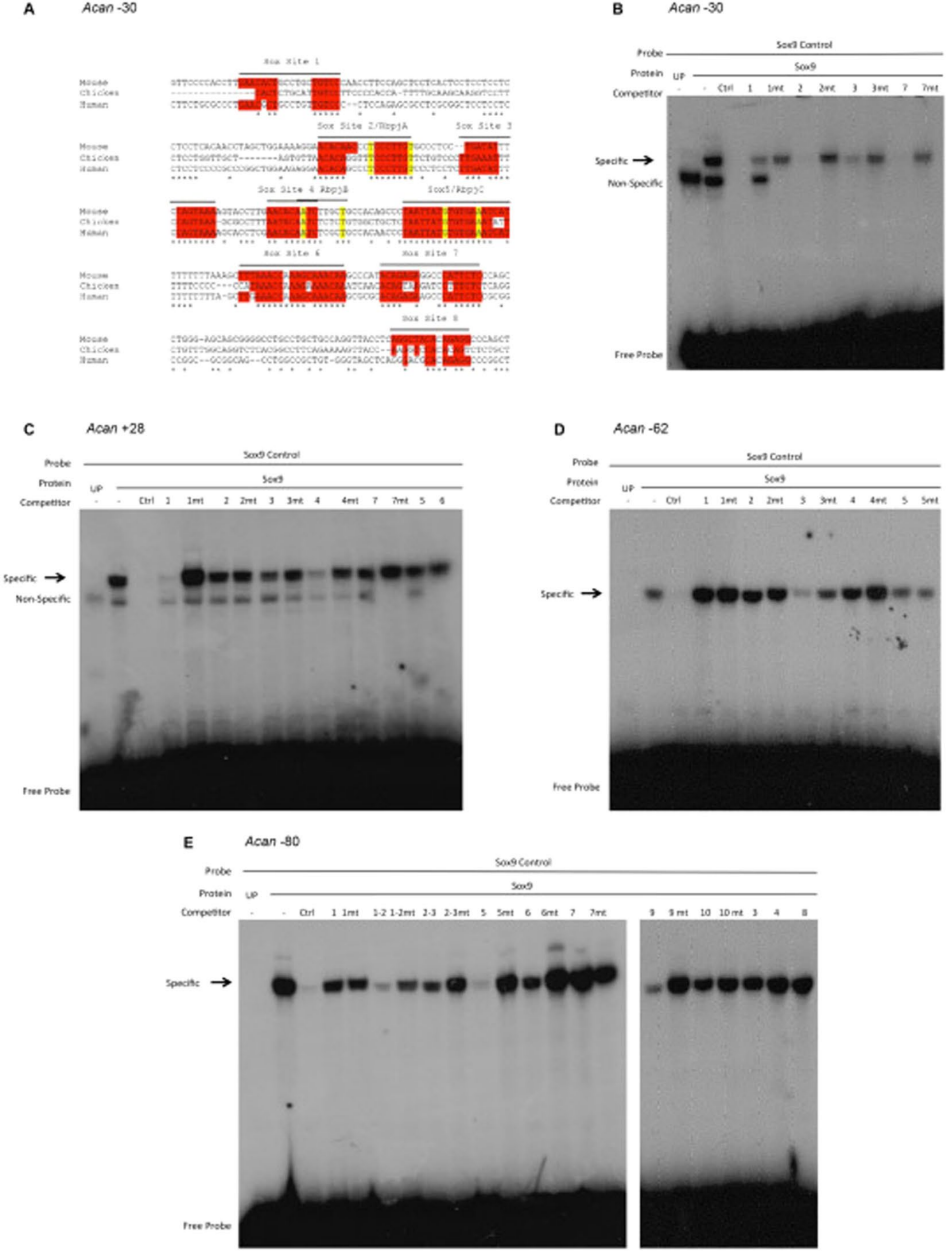
SOX9 indirect competitive Electromobility shift assays. (**A**) ClustalW alignment of the −30 enhancer with possible SOX9 binding sites highlighted in red, the Rbpj-κ binding sites are also highlighted by flanking yellow markings, and each site is identified by the name assigned to each region. SOX9 is predicted to interact through SOX9 dimeric and monomeric interactions in the −30 and all *Acan* enhancer. (**B**) Indirect competitive shift EMSAs using radiolabelled control of the *Col2a1* SOX9 binding site probe incubated with SOX9 protein generated from a SOX9 expression vector, UP is a programmed lane generated by no expression vector to detect any non-specific binding, Ctrl is the unlabelled control competing with the labelled probe to ensure competition is detected, (mt = mutated). Wildtype probes from the −30 sites 2, 3 and 7 competed away the binding of the SOX9 control oligo that suggests these sites bind to SOX9. (**C**) The +28 has two SOX9 binding sites (1 and 4) although they are weaker than the control oligo. (**D**) The −62 contains one site (site 5) and the −80 encloses three binding sites, 1-2, 5 and 9 (**E**). The −80 EMSA consisted of two gels ran simultaneously to allow interrogation of all the possible SOX9 binding sites with mutated oligonucleotides alongside.

The mouse *Acan* enhancer regions show significant sequence conservation in mammals but poor sequence homology in reptiles and fish. We used BioBase and ClustalW analysis to assess the transcription factors that could bind and drive chondrocyte enhancer activity. We first screened the *Acan* enhancer sequences for transcription factor binding sites of the chondrocyte master regulator SOX9, as it is required for the chondrocyte fate, and was shown before to drive activity of A1 enhancer at −10 kb^20^. Screening using BioBase and the conserved enhancer sequences revealed multiple SOX9 binding motifs of the consensus [A/T] [A/T] CAA [A/T] G^28,29^ and in the characteristic adjacent sequences with each SOX9 arranged in opposite orientation separated by 3-4 bp within the enhancers (Fig. 4A site 2, 3 and 7). The only exception was the −80 region, in which there were additional predicted SOX9 binding motifs that do not follow the paired SOX9 binding arrangement (Supplementary Figure S5). We then conducted indirect competitive EMSAs for each of these sites. This was a quick method for screening if these regions could compete with the *Col2a1* SOX9 binding sequence as a control, when incubated in excess (Supplementary Table S2).

The −30 sequence contained three sites, which bound SOX9, namely site 2, 7 and weakly site 3 (Fig. 4B). In the +28 enhancer sequence, SOX sites 1 and 4 weakly out-competed the consensus SOX9 binding sequence probe indicating that they can bind SOX9 protein (Fig. 4C and Figure S3). The −62 region showed interaction of SOX9 at site 3 (Fig. 4D and Figure S4). The −80 region appears to be split into two SOX9 interacting sequences with ten predicted binding motifs; only three sites bound to SOX9 in EMSA experiments (sites 1, 2 and 5, Figure S5) and weakly to site 9 (Fig. 4E). Mutations in SOX binding sites resulted in a lack of binding.

**Figure 5:**
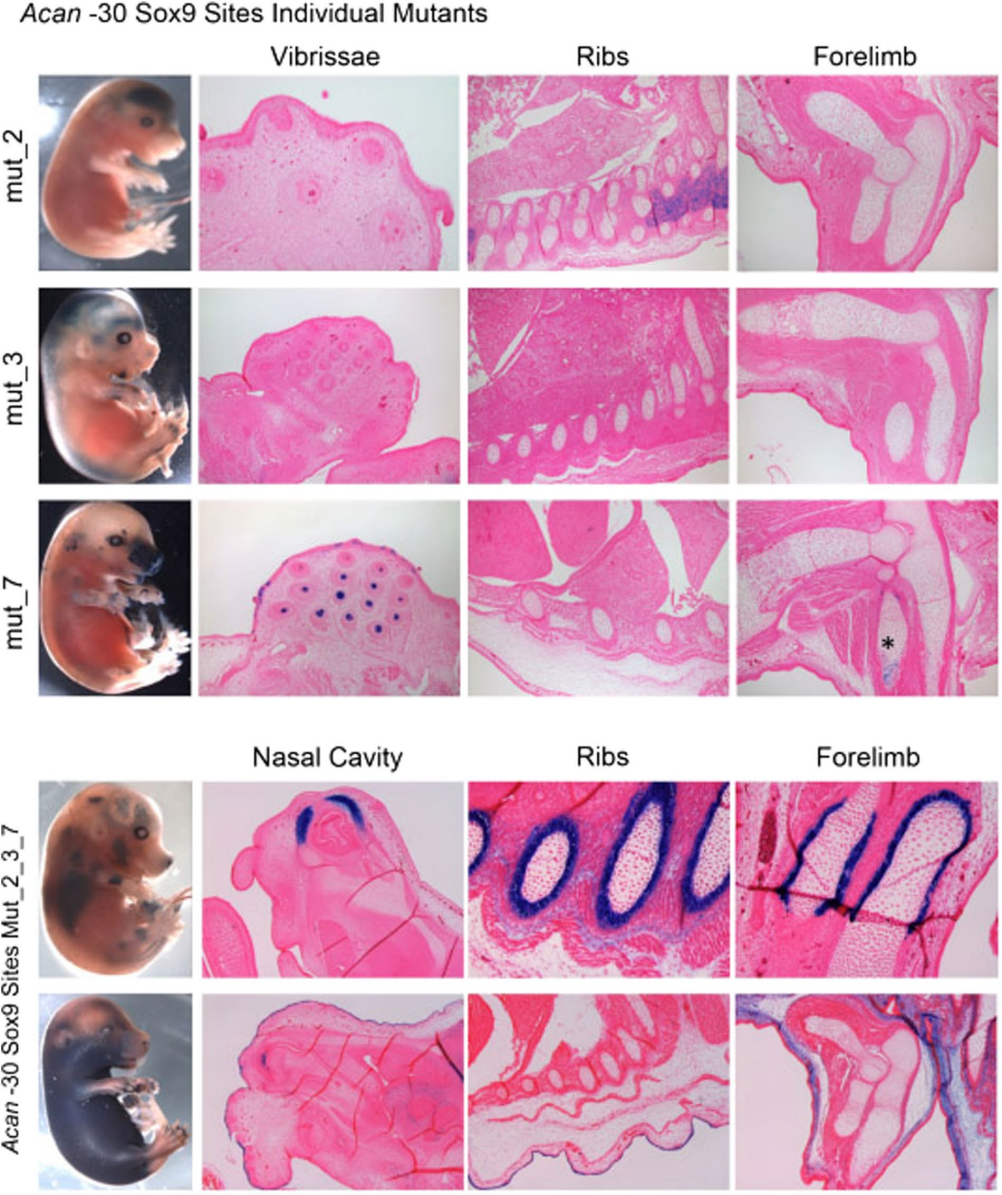
*In vivo* analysis of SOX9 binding site mutations in the −30 enhancer at E15.5. (Top) Individual mutations of the SOX9 binding sites of the −30, Site 2 by itself remove the chondrocyte specificity and some neural activity is detected. Site 3 presented two activity patterns; in both cases chondrocyte specific activity is abolished. In site 7 some chondrocyte activity is detected in the forelimb (*), but mostly all chondrocyte activity is diminished. (Bottom) SOX9 binding site mutations for sites 2, 3 and 7 of the −30 sequence were introduced into transgenic mice. Together they abolish the chondrocyte specific activity of the enhancer. But active in other cells types, the perichondrium and dermal fibroblasts are seen, the nasal cavity in both cases show no staining in the maxilla and some ectopic staining in the mucus membrane.

To determine the importance of SOX9 in driving the activity of the *Acan* enhancers *in vivo*, we generated new transgenic mice harbouring the same mutated sequences (Supplementary data) used in the EMSA experiments of the −30 sequence. We chose this enhancer because it was the most potent and it is active in all chondrocytes in both development and adulthood. Mutations generated in each of the individual SOX9 sites independently abrogated the activity of this enhancer in chondrocytes (Fig. 5). When all SOX9 sites were mutated, the loss of chondrocyte staining was accompanied by a shift in the expression of the transgene in dermal fibroblast or perichondrium localisation in mice at E15.5 (Fig. 5).

This suggested that each site is important for the specificity and robustness of the enhancer in 3D space relative to each other, the removal of all the SOX9 binding sites may result in exposure or create binding sites of other factors binding sites or removes the competition between SOX9 and other factors.

### Rbpj-κ is required for the chondrocyte specificity in development

Notch signalling activates SOX9 to differentiate chondrocyte lineage of mesenchymal cells^30–32^. Therefore, we next examined the enhancer sequences for binding sites for Rbpj-κ, [C/T] GTG [G/A] GAA, a transcriptional regulator involved in Notch signalling that has been previously shown to be required for the onset of hypertrophy and chondrocyte maturation at E14.5^30–32^.

In the −30 sequence, three sites were identified as potential Rbpj-κ binding sites (Fig. 4A) of which only in site C Rbpj-κ appears to bind (Figure S2A)*. In vivo*, all embryos containing mutated versions of the binding site expressed the transgene sporadically in chondrocytes and in some cases, activity was found in dermal fibroblasts (Supplementary Figure S2B), suggesting that Rbpj-κ may, along with SOX9, achieve global expression in chondrocytes.

## Discussion

Aggrecan is an essential component of cartilage not only because the GAG-attachment region provides the high anionic charge density that is needed for the unique ‘hydraulic shock absorber’ properties of cartilage^12,33^. It also appears to protect the cartilage by shielding the collagen type II network as demonstrated by studies involving mutant aggrecan and ADAMTS knock out mouse models^34,35^. In the presence of aggrecan the collagen network in the articular cartilage cannot be cleaved. Therefore, maintaining high levels of aggrecan in cartilage is essential to the survival and integrity of the cartilage. In order to understand what drives the expression of aggrecan in chondrocytes, we investigated potential enhancers that drive high expression of the aggregan gene. Enhancers are *cis*-acting DNA sequences that, in combination with transcription factors, drive the expression of a gene in a particular cell lineage^36–38^. The activity of the transgene driven by each of the four enhancers characterised in this study showed a spatial and temporal distribution of this gene in different types of cartilage. Although all enhancers were active in chondrocytes, the repertoire of activity varied in chondrocyte location and pattern suggesting that multiple enhancers within *Acan* are not merely duplication of activity.

The data presented here suggest that there are two types of regulatory function for these enhancer sequences. The first is a redundant function to allow robust expression of a complex critical gene; this can be seen in the similarity of activity between +28 and −30 kb in all cartilage regions in addition to the already reported −10 kb^20^. However, while these three enhancers drive the expression of the transgene in fibro-, elastic and hyaline cartilages during development, the transgene activity driven the −10 enhancer remains active in adult discs. The second regulatory function, is that enhancers can provide the signals to dictate how chondrocytes build cartilage tissue in different locations by engaging different enhancers and *trans* acting factors during development. As such the −62 enhancer drives reporter activity primarily in late-proliferating and pre-hypertrophic chondrocytes of the limbs and ribs but not in other chondrocyte types. Moreover, differential transgene activity between development and adult tissue is yet another example. The −80 enhancer shows low activity in embryos but is highly active in adult chondrocytes. These patterns of enhancer activities provide us with a roadmap, which would enable further studies for each of these enhancers to illustrate how these sequences may act synergistically or independently to coordinate the expression of the gene and build the intricate different ECM that is required in different types of cartilage. One limitation of this study, is that we did not generate knock-out models for each of the enhancer elements in order to assess the expression levels of ACAN itself.

Since SOX9 is the master regulator of chondrocyte fate, it is not surprising that binding motifs were found within these *Acan* enhancers. In addition, the *Acan* locus appears to interact with SOX9 frequently when examined by ChIP data from other reported studies^23–25^. Our results of *in vitro* investigation using EMSA identified SOX9 binding to all four enhancers, suggesting that their chondrocyte specificity is conferred by this factor. To validate the importance of SOX9 in regulating the *Acan* enhancers, we chose to mutate the binding sites in the −30 element since this enhancer expressed in all chondrocyte in development and adulthood. When mutations were introduced in each of the three dimeric SOX9 binding sites independently there was a loss of transgene activity in chondrocyte in sites 2 and 3 and to a large extent with site 7. When comparing the classical SOX9 binding sequence^20^ with the sites indicated in −30 sequence, they appeared to be weak binder of SOX9, yet a mutation in one sequence impaired global transgene activity in cartilage, suggesting that all three were needed to maintain enhancer activity. We speculate that the requirement of three sites allow the remodelling of the chromatin to initiate transcription with the loss of any of the three sites the remodelling cannot occur so the chondrocyte specific activity is lost^39^. What is interesting is when all three SOX9 sequences were mutated; transgene activity was abolished from all chondrocytes and shifted to either periosteum or fibroblastic cells. This result supports the idea that SOX9 is required for chondrocyte specificity and it may repress the expression of non-chondrogenic cell lineages. Moreover, the shift indicates that other transcription factors are able to regulate the activity of the −30 enhancer in different tissues, only when SOX9 is not bound. This is not unusual since in other cell lineages the binding of SOX9 blocks differentiation into other cell types such as in osteoblasts by inducing β-catenin signalling^40–42^. Moreover, in vascular smooth muscle cells (vSMC), the TF Jagged 1 (Jag1) represses SOX9 to retain the vSMC fate, whereas a loss of Jag1 resulted in ectopic cartilage nodules in the vascular wall of Jag1 smooth muscle knock-out mice at E18.5^43^. Our data also show that while SOX9 is essential for Acan enhancers chondrocyte activity, other factors may stabilise this interaction and we show that *Rbpj-κ* is able to bind −30 enhancer. We selected *Rbpj-κ* because it is required for terminal differentiation and regulates endochondral ossification and is necessary for the maintenance of joint and articular cartilage^44–47^. We suggest that Rbpj-κ can co-regulate the expression of *Acan* along with SOX9 as knockout of Rbpj-κ resulted in a reduction of *Acan* mRNA^48^.

The data presented here gives a comprehensive analysis of multiple *cis*-acting sequences that regulate *Acan* gene in a mammalian system. It gives us the first insights into how different enhancers are active in different types the chondrocytes during development and in adult tissue. These enhancers may be used to address the production of *Acan* in normal cartilage physiology. They might also prove to be key targets to combat diminishing *Acan* in ageing and connective tissue pathologies. In debilitating diseases such as osteoarthritis, where increased mechanical stress on chondrocytes may lead to chondrocyte loss of proper ECM synthesis, these enhancers may be targets to recover Acan production in these diseases.

The characterisation of the *Acan* enhancers offers insights into the molecular mechanism for matrix deposition and how extracellular matrix maintenance can be regulated by different stimuli. The emergence of CRISPR-cas9 genome editing offer new tools for studying the maintenance of cartilage biology and in pathologies.

## Methods

### Identification of limb specific aggrecan enhancers

The University of California at Santa Cruz (UCSC) genome browser (https://genome.ucsc.edu/) mouse assembly July 2007 mm9 (NCBI37/mm9) was used to identify sequences up to 100 kb upstream of *Acan*. Possible enhancer elements were selected based on the presence of the enhancer-associated histone modifications mono-methylation of histone 3 lysine 4 (H3K4me1), acetylation of histone 3 lysine 27 (H3K27ac) and tri-methylation of histone 3 lysine 4 (H3K4me3) in E14.5 mouse limbs detected by Chromatin immunoprecipitation followed by sequencing (ChIP-seq) found in the ENCODE/ Ludwig Institute of Cancer Research (LICR) project track. In addition, high conservation throughout vertebrate genomes and the presence of DNase I hypersensitivity in Embryonic day 11.5 (E11.5) mouse limb buds, NIH3T3 cells and fibroblasts from the ENCODE project University of Washington were used as criteria for enhancer elements.

### Cloning of *Acan* and mutant enhancers constructs

Enhancer sequences were generated by PCR from mouse genomic DNA using primers designed with flanking 100 bp 5′ and 3′ (Supplementary Table S2) using the REDExtract-N-Amp™ PCR Ready Mix™ (Sigma). Fresh PCR products were cloned into the pCR8/GW/TOPO vector using the pCR8/GW/TOPO TA Cloning Kit (Invitrogen) by incubation at 25 °C for 30 minutes and transformed into One Shot® TOP10 Chemically Competent *E. coli* (Invitrogen).

Mutant versions of the enhancers were initially generated as custom-made, double-stranded linear DNA fragments (GeneArt® Strings™, Life Technologies) and were treated as PCR products as described above. Once successful cloning was confirmed by restriction enzyme digestion, the enhancer sequences were transferred from the pCR8/GW/enhancer entry vector to the HSP68-*LacZ-*Gateway® destination vector^26^ using Gateway LR Clonase II Enzyme mix (Life Technologies) following manufacturer’s instructions.

### Generation and analysis of transgenic mice

All animal procedures were approved by local ethical review and licensed by the UK Home Office (70/7288). All methods were performed in accordance with guidelines and regulations outlined by the UK Home Office. Transgenic mice were generated by oocyte microinjection as described previously^49^. Mice were housed in the specific pathogen free (SPF) animal unit at the University of Liverpool.

Transgenic mouse embryos were collected at embryonic day 15.5 (E15.5) and tissue collected from 8-week-old mice. After dissection, samples were rinsed in ice-cold 1 × PBS and fixed in 0.2% glutaraldehyde, 0.1 M sodium phosphate buffer, 5 mM Ethylene glycol-bis (2-aminoethylether)-*N, N, N’, N’*-tetraacetic acid, 2 mM MgCl_2_ and 2% formalin.

After fixation and tissue were rinsed in 0.1M sodium phosphate buffer, 2 mM MgCl_2_, 0.1% sodium deoxycholate and 0.2% Nonidet P-40 Substitute (Sigma) and then stained overnight at room temperature in 1 mg/ml 5-bromo-4-chloro-3-indolyl-β-D-galactoside solution (X-gal) containing 5 mM potassium ferrocyanide, 5 mM potassium ferricyanide, 0.1% sodium deoxycholate, 0.2% Nonidet P-40 Substitute, 2 mM MgCl_2_ and 1 × PBS. After staining, embryos were rinsed through a series of washes, and then fixed overnight in 10% neutral buffered formalin at 4 °C. Eight weeks old mice bones were decalcified using 10% EDTA for two weeks prior to tissue processing.

Imaging of whole embryos was performed using the Olympus SZX12 equipped with the Qimaging QIClick™ CCD camera and QCapture Pro software. For each enhancer, embryos were also sectioned for histological analysis to investigate localisation of X-gal staining. For histological analysis, embryos were dehydrated through a series of ethanol washes, cleared by xylene and paraffin wax-embedded. 6-μm sections were prepared, de-waxed, and counterstained with Eosin (Leica). Eosin counter-stained sections were acquired on an Olympus BX60 using the CellD software. Images were collated and processed using Corel Paintshop Pro X6 and Adobe Photoshop CC 2017. Original images were only adjusted to size and aligned with no manipulation of brightness, contrast or direct changes to the image itself.

Transgenic lines were created for each of the expressing *cis*-acting elements. Ear notches of adult mice and tail samples from embryos were used for genotyping. Samples were digested in lysis buffer (50 mM Tris-HCL pH8, 0.1 M NaCl, 1% SDS, 20 mM EDTA) with proteinase K (10 mg/μl) and ethanol precipitated. 150 ng/μL of DNA was used for PCR using the GoTaq®G2 Flexi DNA polymerase (Promega) supplemented with 2.5 mM MgCl_2_ using *LacZ* specific primers (Supplementary Table S2).

### Identification of transcription factor binding sites

Each sequence was aligned using ClustalW^50^ and subjected to analysis using the Transfac software (BioBase)^51^ and conservation for the most represented sites.

### Electrophoretic mobility shift assay

Proteins for indirect competition Electrophoretic mobility shift assays (EMSAs) were made using the TNT T7/SP6 coupled reticulocyte lysate system (Promega) for SOX9 using 4XFLAG-tagged SOX9 expression plasmid^52^ and recombination signal binding protein for Ig kappa J (Rbpj-κ) from pcDNA3.1 plasmid using T7 RNA polymerase following manufacturer’s instructions. Unprogrammed protein was generated by replacing the expression vector with an empty vector.

Double stranded oligonucleotides were labelled with α−32P dCTP (3000 Ci/mmol 10mCiml, 250μCi) (Perkinelmer) using Klenow (Promega) to fill in overhanging 5′ ends and purified on non-denaturing acrylamide gel. 20 μL binding reactions consisted of 2 μg protein, 0.48 μg of poly (dG-dC) and 2 μL 10× binding buffer (40 mM KCl, 15 mM HEPES pH 7.9, 1 mM EDTA, 0.5MM DTT, 5% glycerol). 100-fold excess (1ug/mL) or increasing concentration (0.4, 0.6 or 0.8ug/mL of competitor oligonucleotides was added to the binding reaction in a volume of 1 μL and incubated at 30 °C for 20 minutes. Radiolabelled probes (20,000 counts per minutes (CPM)) were added and incubated for a further 40 minutes. Gels were electrophoresed on an 18 × 16 cm 6% non-denaturing polyacrylamide gel at 4 °C for 2 hours in the Hoefer™ SE400 Air-cool vertical electrophoresis system. Gels were dried and exposed to X-ray films for 72 hours. Films were scanned as a 600dpi image and collated on Adobe Photoshop CC 2017. Original gel images (Supplementary Figures S6 and S7) were cropped and aligned. For Fig. 4E two gels were ran and processed simultaneously to allow for all the oligonucleotides probes to be examined for the enhancer concurrently.

### Immunohistochemistry

The BR-1 primary antibody was generated by Professor Tim Hardingham. It is a polyclonal rabbit anti-pig antibody raised against the G1 domain of Aggrecan. Slides containing sections from the −10 kb and −80 enhancer transgenic at E15.5 were deparaffinised by sequential incubation in xylene, 100% ethanol, 95% ethanol and 75% ethanol. Antigen retrieval was performed using Bovine testicular hyalouronidase (1000U/ml in PBS, Sigma) at 37 °C for 1 hour. Endogenous peroxidase activity was blocked using 0.3% hydrogen peroxidase. Primary antibody was incubated overnight at 4 °C at a dilution of 1:1000 in Dako Antibody Diluent. After three 5 minutes washes in PBS, slides were incubated at room temperature with peroxidase conjugated Goat anti-Rabbit (Santa Cruz Biotechnology) for 30 minutes. Peroxidase (Dako) substrate was added for 30 seconds, slides were counter stained with Harris haematoxylin (Lieca) and coverslipped using Histomount (National Diagnostics).

## Electronic supplementary material

Supplementary Information
