# Supplementary material for: Differential tissue specific, temporal and spatial expression patterns of the Aggrecan gene is modulated by independent enhancer elements
Source: Sci Rep. 2018 Jan 17;8:950. doi: 10.1038/s41598-018-19186-4 (PMC5772622; doi:10.1038/s41598-018-19186-4)
Supplement: Supplementary file 1 — Supplementary Information [file 41598_2018_19186_MOESM1_ESM.doc]

**Differential tissue specific, temporal and spatial expression patterns of the Aggrecan gene is modulated by independent enhancer elements**

Ian M H Li, Ke Liu, Alice Neal, Peter D Clegg, Sarah De Val and George Bou-Gharios

**Supplementary Data**

**Table S1: Summary of staining intensities and location of *Acan* enhancers at E15.5.**

- Is not detected, + weakly active, ++ not completely uniformly active in the tissue/cell, +++ majority of tissue activity,
++++ strong staining uniformly throughout tissue, ± active in some founder embryos not all. uDenotes activity was seen only in hypertrophic chondrocytes.

**PCR and EMSA primers**

**Table S2:** Primers used to create enhancer reporter constructs and oligos for EMSA experiments. Underlined indicates non-homologues 5’ tag to permit radioisotope labelling. All oligos were obtained from Eurofins Genomics (Germany).

| **Oligo Name** | **Oligo Sequence** | |
| --- | --- | --- |
| *LacZ* PCR Primer F | 5’-GTTGCAGTGCACGGCAGATACACTTGCTGA-3’ | |
| *LacZ* PCR Primer R | 5’-GCCACTGGTGTGGGCCATAATTCAATTCGC-3’ | |
| *Acan* +28 F | 5’-CTGAGTCTTGACAGAGCTGCTA-3’ | |
| *Acan* +28 R | 5’-GATTGGTCAGTAGTGGAGCACA-3’ | |
| *Acan* +28 Small F | 5’-TCCATCTCAGGCCTTCCTCT-3’ | |
| *Acan* +28 Small R | 5’-AGGGCATAGAGGCACATGAC-3’ | |
| *Acan* A1 F | 5’- GGT GAC ACC CAA ACT AAC AGC-3’ | |
| *Acan* A1 R | 5’- ATC TCA CAC GTG GGC ATT TGC-3’ | |
| *Acan* -30 F | 5’- GGCTAGACCAGTAGAACCCA-3 | |
| *Acan* -30 R | 5’-TGCATCATTACAGTCTTTCTTCAG -3 | |
| *Acan* -30 Small F | 5’-CGCATGCACACCGCCCTCCT-3’ | |
| *Acan* -30 Small R | 5’-CTTCAGCAGTCAGGCAGGGGA-3’ | |
| *Acan* -62 F | 5’-ATGTTCAGACAGCTCCAACCC-3’ | |
| *Acan* -62 R | 5’-GGGTTGTGTTAATAGGCACGG-3’ | |
| *Acan* -80 F | 5’-CAGACTGTATTTCCCGAGTACCT-3’ | |
| *Acan* -80 R | 5’-TGGAAGAGGGGCAAAATGAGA-’3 | |
| *Acan* -80 Small F | 5’-GTGAGGACTGAAGGGAACGA-3’ | |
| *Acan* -80 Small R | 5’-CCTGGGACAAGGTTTTGTGG-3’ | |
| *Sox9* control F | CTAGGATCCAAAGCCCCATTCATGAGATCTG | |
| *Sox9* control R | CTAGCAGATCTCATGAATGGGGCTTTGGATC | |
| *Rbpj-k* control F | ggggtgaggtctatttcccacgacatacttcc | |
| *Rbpj-k* control R | gggggaagtatgtcgtgggaaatagacctcac | |
| *Acan* -30 Sox 1F | CTAGCTTGAACACTGCCTGCTGTCCCAACC | |
| *Acan* -30 Sox 1R | CTAGGGTTGGGACAGCAGGCAGTGTTCAAG | |
| *Acan* -30 Sox 1mut F | CTAGCTTCCTGACTGCCTGCACCCCCAACC | |
| *Acan* -30 Sox 1mutR | CTAGGGTTGGGGGTGCAGGCAGTCAGGAAG | |
| *Acan* -30 Sox 2F | CTAGAAAGGAACACAACCCTCCCTTGTGCCCTCCT | |
| *Acan* -30 Sox 2R | CTAGAGGAGGGCACAAGGGAGGGTTGTGTTCCTTT | |
| *Acan* -30 Sox 2mutF | CTAGAAAAGGGGCTCCGCCCTCCAGGGAGCCCTCCT | |
| *Acan* -30 Sox 2mutR | CTAGAGGAGGGCTCCCTGGAGGGCGGAGCCCCTTTT | |
| *Acan* -30 Sox 3F | CTAGGTGCCCTCCTGATATTTCCAGTAAAAAGTACCTT | |
| *Acan* -30 Sox 3R | CTAGAAGGTACTTTTTACTGGAAATATCAGGAGGGCAC | |
| *Acan* -30 Sox 3mutF | CTAGGTGCCCTCCTTGGGCCTCCCGTCCACGTACCTT | |
| *Acan* -30 Sox 3mutR | CTAGAAGGTACGTGGACGGGAGGCCCAAGGAGGGCAC | |
| *Acan* -30 Sox 4F | CTAGCCTTGAACACAATCTTGCTGCCACAGCCCTAA | |
| *Acan* -30 Sox 4R | CTAGTTAGGGCTGTGGCAGCAAGATTGTGTTCAAGG | |
| *Acan* -30 Sox 4mutF | CTAGCCTTGAGCGCGATCTTGCCGCCACAGCCCTAA | |
| *Acan* -30 Sox 4mutR | CTAGTTAGGGCTGTGGCGGCAAGATCGCGCTCAAGG | |
| *Acan* -30 Sox 5F | CTAGCCCTAATTATGTGTGAAATCATTTTTTTTAAAGC | |
| *Acan* -30 Sox 5R | CTAGGCTTTAAAAAAAATGATTTCACACATAATTAGGG | |
| *Acan* -30 Sox 5mutF | CTAGCCCTAATTATGTGGGCCCTCATTTTTTTTAAAGC | |
| *Acan* -30 Sox 5mutR | CTAGGCTTTAAAAAAAATGAGGGCCCACATAATTAGGG | |
| *Acan* -30 Sox 6F | CTAGAAAGCTTTAAACCAAAGCAACAAGCCCTA | |
| *Acan* -30 Sox 6R | CTAGTAGGGCTTGTTGCTTTGGTTTAAAGCTTT | |
| *Acan* -30 Sox 6mutF | CTAGAAAGCTCTGGCCCAACGCACACAGGCCCAT | |
| *Acan* -30 Sox 6mutR | CTAGATGGGCCTGTGTGCGTTGGGCCAGAGCTTT | |
| *Acan* -30 Sox 7F | CTAGCCCATACAGAGAGGCCCATTCTCCCAGCCC | |
| *Acan* -30 Sox 7R | CTAGGGGCTGGGAGAATGGGCCTCTCTGTATGGG | |
| *Acan* -30 Sox 7mutF | CTAGGGCTGGGAGCCTGGGCCCCTCCGTATGGG | |
| *Acan* -30 Sox 7mutR | CTAGGGCTGGAGCCTGGGCCCCTCCGTATGGG | |
| *Acan* -30 Sox 8F | CTAGGGTTACCTCAGGCTACACAGAGGCC | |
| *Acan* -30 Sox 8R | CTAGGGCCTCTGTGTAGCCTGAGGTAACC | |
| *Acan* -30 Sox 8mutF | CTAGGGTTACCTCCTCCTACTTGGAGCGCC | |
| *Acan* -30 Sox 8mutR | CTAGGGCGCTCCAAGTAGGAGGAGGTAACC | |
| *Acan* -30 Rbpj-A F | CTAGACACACCCTCCCTTGTGCCCTCCT | |
| *Acan* -30 Rbpj-A R | CTAGTCAGGAGGGCACAAGGGAGGGTGTGT | |
| *Acan* -30 Rbpj-A mutF | CTAGACACAACCCTCGGTTCGGCCTCCTGA | |
| *Acan* -30 Rbpj-A mutR | CTAGTCAGGAGGCCGAACCGAGGGTTGTGT | |
| *Acan* -30 Rbpj-B F | CTAGACCTTGAACACAATCTTGCTGCC | |
| *Acan* -30 Rbpj-B R | CTAGGGCAGCAAGATTGTGTTCAAGGT | |
| *Acan* -30 Rbpj-B mutF | CTAGACCTTGAACGTGGCTTGCTGCC | |
| *Acan* -30 Rbpj-B mutR | CTAGGGCAGCAAGCCAACGTTCAAGGT | |
| *Acan* -30 Rbpj-C F | CTAGCCTAATTATGTGTGAAATCATTTTTT | |
| *Acan* -30 Rbpj-C R | CTAGAAAAAATGATTTCACACATAATTAGG | |
| *Acan* -30 Rbpj-C mutF | CTAGCCTAATTATTTGTTAAATCATTTTTT | |
| *Acan* -30 Rbpj-C mutR | CTAGAAAAAATGATTTAACAAATAATTAGG | |
| *Acan* -80 Sox 1F | CTAGGGAACGACAAAATGTCCT | |
| *Acan* -80 Sox 1R | CTAGAGGACATTTTGTCGTTCC | |
| *Acan* -80 Sox 1mF | CTAGGGAACGTCGGAATGTCCT | |
| *Acan* -80 Sox 1mR | CTAGAGGACATTCCGACGTTCC | |
| *Acan* -80 Sox 1_2F | CTAGGGAACGACAAAATGTCCTTACTGTCCCCAC | |
| *Acan* -80 Sox 1_2R | CTAGGTGGGGACAGTAAGGACATTTTGTCGTTCC | |
| *Acan* -80 Sox 1_2mF | CTAGGGAACGACATTATGTCCTTACTGGACCCAC | |
| *Acan* -80 Sox 1_2mR | CTAGGTGGGTCCAGTAAGGACATAATGTCGTTCC | |
| *Acan* -80 Sox 2_3F | CTAGGTCCTTACTGTCCCCACTTGTGGTCCA | |
| *Acan* -80 Sox 2_3R | CTAGTGGACCACAAGTGGGGACAGTAAGGAC | |
| *Acan* -80 Sox 2_3mF | CTAGGTCCTTCCGGTCCCCACTGGTGGTCCA | |
| *Acan* -80 Sox 2_3mR | CTAGTGGACCACCAGTGGGGACCGGAAGGAC | |
| *Acan* -80 Sox3F | CTAGCCCCACTTGTGGTCCAATCTGCCAAC | |
| *Acan* -80 Sox3R | CTAGGTTGGCAGATTGGACCACAAGTGGGG | |
| *Acan* -80 Sox3mF | CTAGCCCCACTGGGGGTCCATTCTGCCAAC | |
| *Acan* -80 Sox3mR | CTAGGTTGGCAGAATGGACCCCCAGTGGGG | |
| *Acan* -80 Sox4F | CTAGGAGAGTACATTTCCACGAGGC | |
| *Acan* -80 Sox4R | CTAGGCCTCGTGGAAATGTACTCTC | |
| *Acan* -80 Sox4mF | CTAGGAGAGTTCCTTTCCACGAGGC | |
| *Acan* -80 Sox4mR | CTAGGCCTCGTGGAAAGGAACTCTC | |
| *Acan* -80 Sox5F | CTAGTCCCAAACAACAGTACGGGC | |
| *Acan* -80 Sox5R | CTAGGCCCGTACTGTTGTTTGGGA | |
| *Acan* -80 Sox5mF | CTAGTCCCAAATTAGGGAACGGGC | |
| *Acan* -80 Sox5mR | CTAGGCCCGTTCCCTAATTTGGGA | |
| *Acan* -80 Sox6F | CTAGCATATTTGTCTTGGCC | |
| *Acan* -80 Sox6R | CTAGGGCCAAGACAAATATG | |
| *Acan* -80 Sox6mF | CTAGCATCGGGTCTTGGCC | |
| *Acan* -80 Sox6mR | CTAGGGCCAAGACCCGATG | |
| *Acan* -80 Sox7F | CTAGAAAGACAGATTTTCCTTTCCC | |
| *Acan* -80 Sox7R | CTAGGGGAAAGGAAAATCTGTCTTT | |
| *Acan* -80 Sox7mF | CTAGAAAGACAGACGATCCACCCCC | |
| *Acan* -80 Sox7mR | CTAGGGGGGTGGATCGTCTGTCTTT | |
| *Acan* -80 Sox8F | CTAGCCAGCCATTGGCCTCCGC | |
| *Acan* -80 Sox8R | CTAGGCGGAGGCCAATGGCTGG | |
| *Acan* -80 Sox8mF | CTAGCCAGCCCGGTTCCTCCGC | |
| *Acan* -80 Sox8mR | CTAGGCGGAGGAACCGGGCTGG | |
| *Acan* -80 Sox9F | CTAGGTGAGGCTTGTTTGGGATGG | |
| *Acan -80* Sox9R | CTAGCCATCCCAAACAAGCCTCAC | |
| *Acan* -80 Sox9mF | CTAGGTGAGGACGGAACGGGATGG | |
| *Acan* -80 Sox9mR | CTAGCCATCCCGTTCCGTCCTCAC | |
| *Acan* -80 Sox10F | CTAGGATGGGCCACAAAACCTTGTCCC | |
| *Acan* -80 Sox10R | CTAGGGGACAAGGTTTTGTGGCCCATC | |
| *Acan* -80 Sox10mF | CTAGGATGGGCAACTTAACATCGTCCC | |
| *Acan* -80 Sox10mR | CTAGGGGACGATGTTAAGTTGCCCATC | |
| *Acan* ­-62 Sox1F | CTAGAGAATGCGTCTTTGCTCGACCA | |
| *Acan* -62 Sox1R | CTAGTGGTCGAGCAAAGACGCATTCT | |
| *Acan* -62 Sox1mF | CTAGAGAATGCGTTGGTGCTCGACCA | |
| *Acan* -62 Sox1mR | CTAGTGGTCGAGCACCAACGCATTCT | |
| *Acan* -62 Sox2F | CTAGCAGGGCAGCATCAGAGGAGCGAGGA | |
| *Acan* -62 Sox2R | CTAGTCCTCGCTCCTCTGATGCTGCCCTG | |
| *Acan* -62 Sox2mF | CTAGCAGGGCAGTATCAGATTAGCGAGGA | |
| *Acan* -62 Sox2mR | CTAGTCCTCGCTAATCTGATACTGCCCTG | |
| *Acan* -62 Sox3F | CTAGGAGCTGGACAAGGGCAGGCTTTGTGCGCAGCG | |
| *Acan* -62 Sox3R | CTAGCGCTGCGCACAAAGCCTGCCCTTGTCCAGCTC | |
| *Acan* -62 Sox3mF | CTAGGAGCTGTACCAGGGCAGGCGGTGTGCGCAGCG | |
| *Acan* -62 Sox3mR | CTAGCGCTGCGCACACCGCCTGCCCTGGTACAGCTC | |
| *Acan* -62 Sox4F | CTAGTGTCTACTCTGAAGGAAAAT | |
| *Acan* -62 Sox4R | CTAGTGTCTACTCTGAAGGAAAAT | |
| *Acan* -62 Sox4mF | CTAGTGTAGACTCTGCCGGAGGAT | |
| *Acan* -62 Sox4mR | CTAGATCCTCCGGCAGAGTCTACA | |
| *Acan* -62 Sox5F | CTAGAGTCATTTCAAGGAATTGAATTTCAAGGAATCCT | |
| *Acan* -62 Sox5R | CTAGAGGATTCCTTGAAATTCAATTCCTTGAAATGACT | |
| *Acan* -62 Sox5mF | CTAGAGTCATTTCTTAAAACTGAAGCTCGGGGAATCCT | |
| *Acan* -62 Sox5mR | CTAGAGGATTCCCCGAGCTTCAGTTTTAAGAAATGACT | |
| *Acan* +28 Sox1F | CTAGGCTTTCCTTCCCATTGAGAAAGGTCTCT | |
| *Acan* +28 Sox1R | CTAGAGAGACCTTTCTCAATGGGAAGGAAAGC | |
| *Acan* +28 Sox1mF | CTAGGCTTTCCTTCCCAGGGAGCTAATTCTCT | |
| *Acan* +28 Sox1mR | CTAGAGAGAATTAGCTCCCTGGGAAGGAAAGC | |
| *Acan* +28 Sox2F | CTAGGAAAGGTCTCTCTGTCCACGAG | |
| *Acan* +28 Sox2R | CTAGCTCGTGGACAGAGAGACCTTTC | |
| *Acan* +28 Sox2mF | CTAGGAAAGGTTTATCTGGCTACGAG |  |
| *Acan* +28 Sox2mR | CTAGCTCGTAGCCAGATAAACCTTTC |  |
| *Acan* +28 Sox3F | CTAGTCCCTATTTTTCTTTACAAC |  |
| *Acan* +28 Sox3R | CTAGGTTGTAAAGAAAAATAGGGA |  |
| *Acan* +28 Sox3mF | CTAGTCCCTATTTCGACATACAAC |  |
| *Acan* +28 Sox3mR | CTAGGTTGTATGTCGAAATAGGGA |  |
| *Acan* +28 Sox4F | CTAGCTGAGAAAACACACACAAACACAAGAGC |  |
| *Acan* +28 Sox4R | CTAGGCTCTTGTGTTTGTGTGTGTTTTCTCAG |  |
| *Acan* +28 Sox4mF | CTAGCTGAGAATCCTCACACGCACACAAGAGC |  |
| *Acan* +28 Sox4F | CTAGGCTCTTGTGTGCGTGTGAGGATTCTCAG |  |
| *Acan* +28 Sox5F | CTAGCAGGGAGAAAAACAGAGCCAGCCCAAA |  |
| *Acan* +28 Sox5R | CTAGTTTGGGCTGGCTCTGTTTTTCTCCCTG |  |
| *Acan* +28 Sox5mF | CTAGCAGGGAGAATATCAGAGCCAGCGCAAA |  |
| *Acan* +28 Sox5mR | CTAGTTTGCGCTGGCTCTGATATTCTCCCTG |  |
| *Acan* +28 Sox6F | CTAGCAAACCCCACAGAGCACCATTGCATC |  |
| *Acan* +28 Sox6R | CTAGGATGCAATGGTGCTCTGTGGGGTTTG |  |
| *Acan* +28 Sox6mF | CTAGCAAACCTGACAGGCTACCATTGCATC |  |
| *Acan* +28 Sox6mR | CTAGGATGCAATGGTAGCCTGTCAGGTTTG |  |
| *Acan* +28 Sox7F | CTAGCAGCTTGTTCAGAGAGCGAGGGAAGAAACA |  |
| *Acan* +28 Sox7R | CTAGTGTTTCTTCCCTCGCTCTCTGAACAAGCTG |  |
| *Acan* +28 Sox7mF | CTAGCAGCTTGACCAGAGAGCGACGGTAGAAACA |  |
| *Acan* +28 Sox7mR | CTAGTGTTTCTACCGTCGCTCTCTGGTCAAGCTG |  |

**
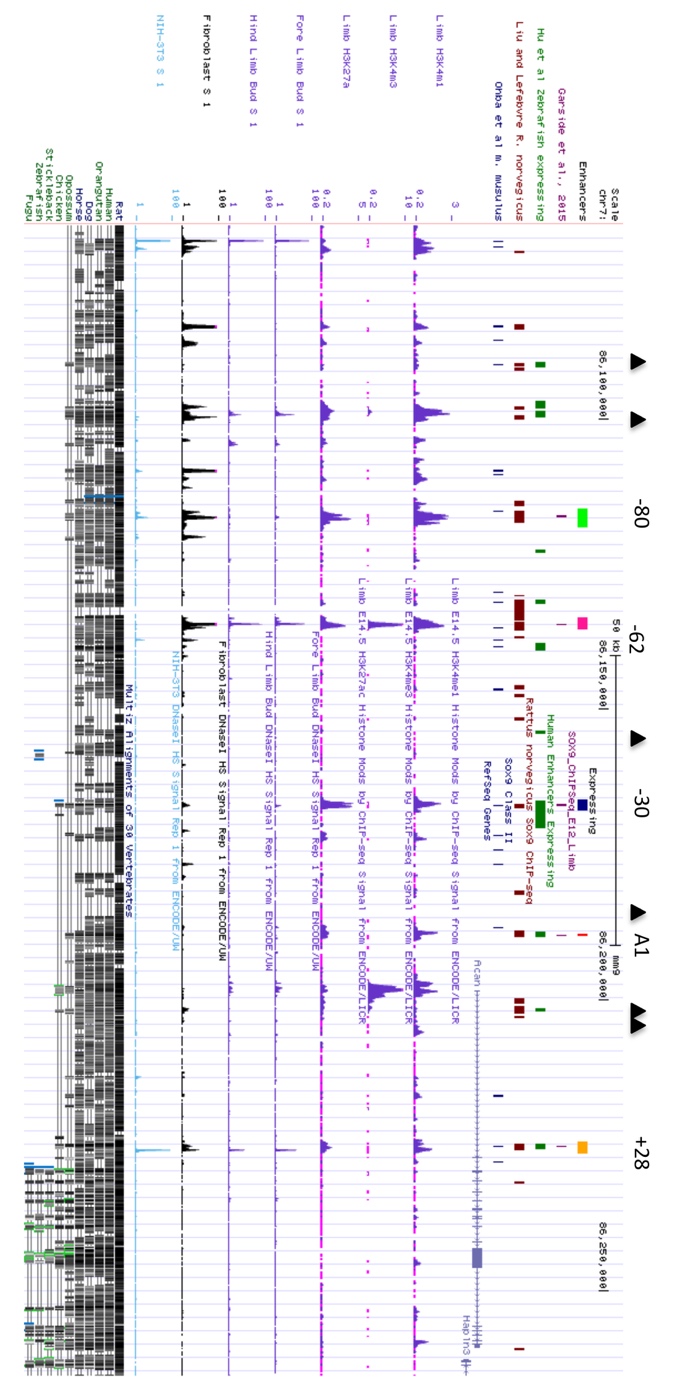
**

**Supplementary Figure S1: Comparison of identified enhancers with other studies.**

Zebrafish enhancer was shown to drive reporter activity similar to Aggrecan expression (Green track), SOX9 ChIP-seq data from three published studies, newborn mouse rib chondrocytes (Blue track) (Ohba et al.,), Rat (Liu and Lefebrve) (Red track) and in mouse limb buds embryonic day 12.5 (E12.5) (Garside *et al.,*) (Purple Track) to ENCODE Histone modification, DNaseI hypersensitivity in fore limb, hindlimb bud, fibroblast and NIH3T3 cells. The enhancers examined in this study are highlighted by their name; +28, -30, -62 and -80. Whereas the known aggrecan enhancer is labelled A1. Black triangles represent additional enhancers that were identified in other studies that were not examined in our study. All the regions we tested bound to SOX9 even in limb buds at E12.5.


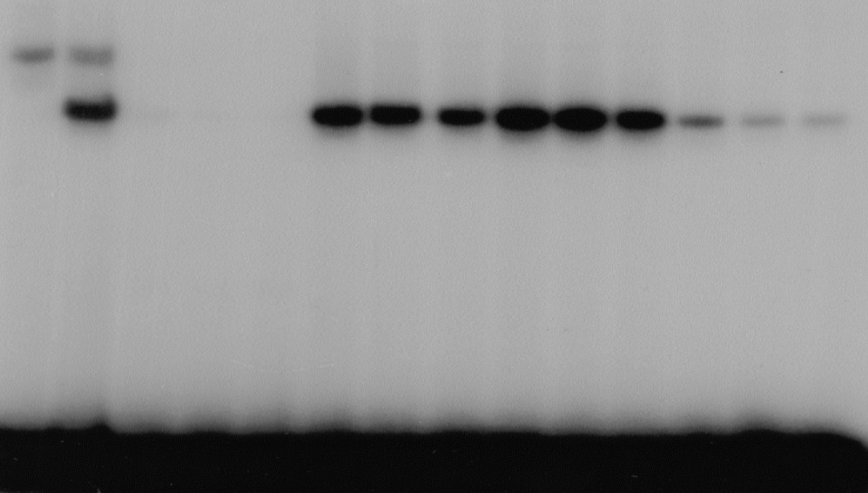


Competitor Concentration

Free Probe

Probe

Non-Specific

RBPJ-K

UP

RBPJ-K Control

-

Ctrl

1

2

3

-

-

Protein

**A**


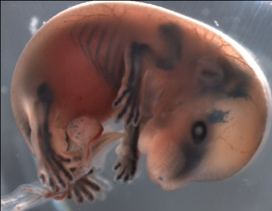

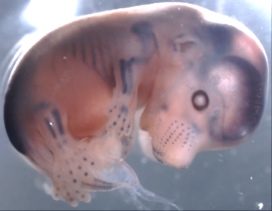

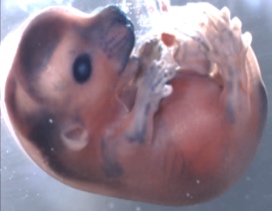

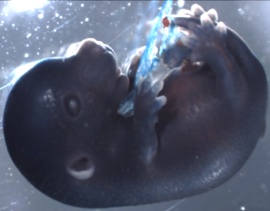

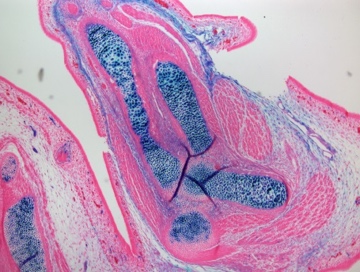

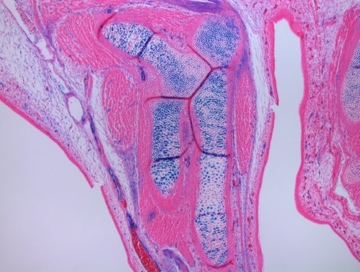

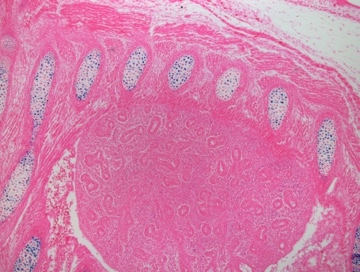

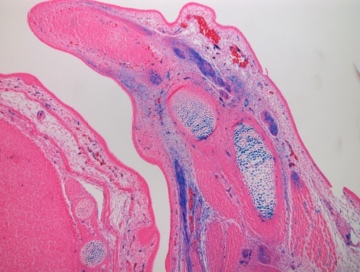

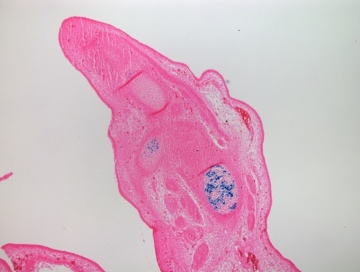

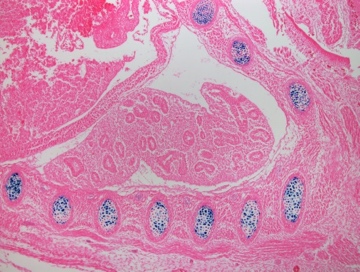

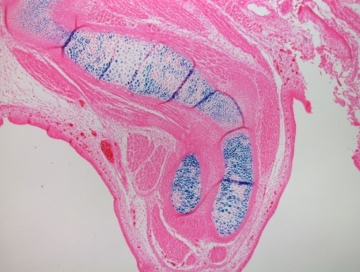

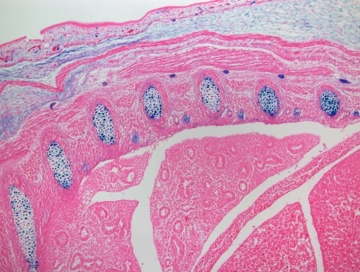

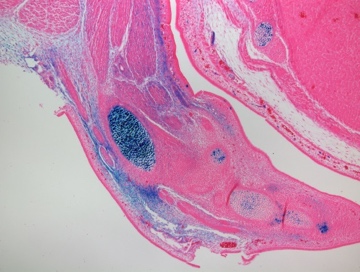


Ribs

Forelimb

Paw

*Acan* -30 Rbpj Site mut_C

**B**

*Acan* -30 Wildtype


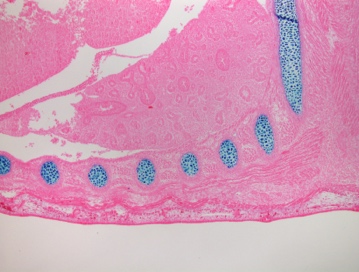

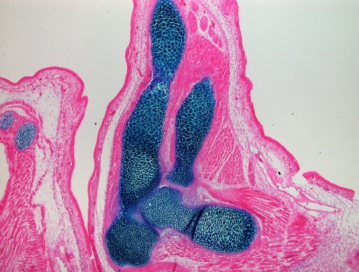

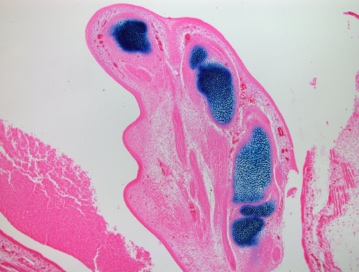


Specific

**Supplementary figure S2: Examination of the role of Rbpj-κ in regulating the -30 enhancer at E15.5. (A)** Indirect competitive EMSAs with increasing concentration of the competitor using the 3 Rbpj-κ sites (A-C) identified in the -30. Site C appears to be binding to Rbpj-κ. **(B)** Introduction of the mutated site C sequence into transgenic mice did not abolish the chondrocyte expression. In most cases the expression is more scattered than the wildtype -30 sequence. There is also an increase in the dermal fibroblast expression. Rbpj-κ may be binding or competing with other transcription factors to prevent the dermal fibroblast expression of the enhancer.

*Acan* +28


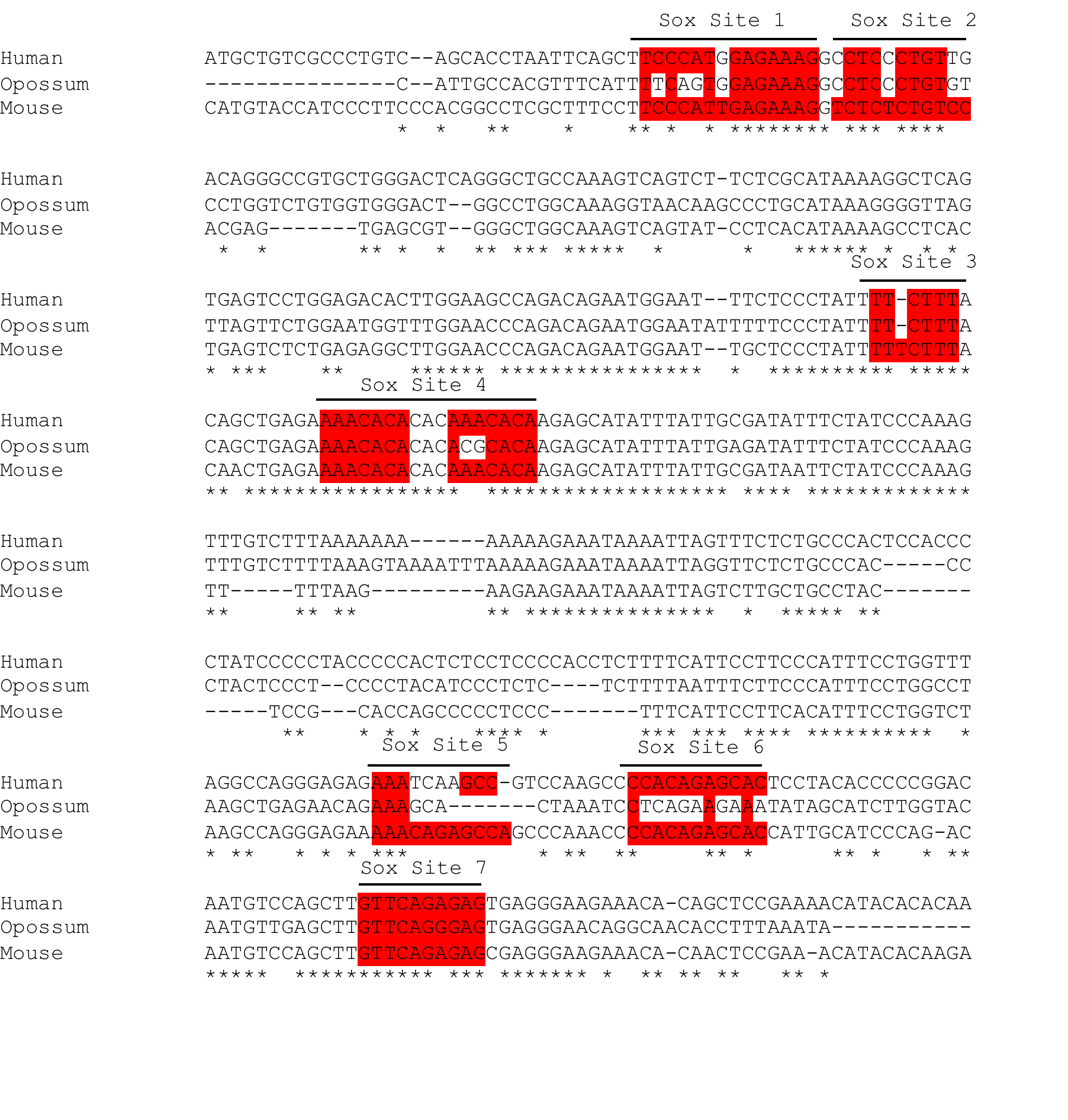


**Supplementary Figure S3:** ClustalW alignment of the +28 enhancer with possible SOX9 binding sites highlighted in red, each site is identified by the name assigned to each region. SOX9 is predicted to interact through SOX9 dimeric and monomeric interactions in the +28 *Acan* enhancer at 7 sites.


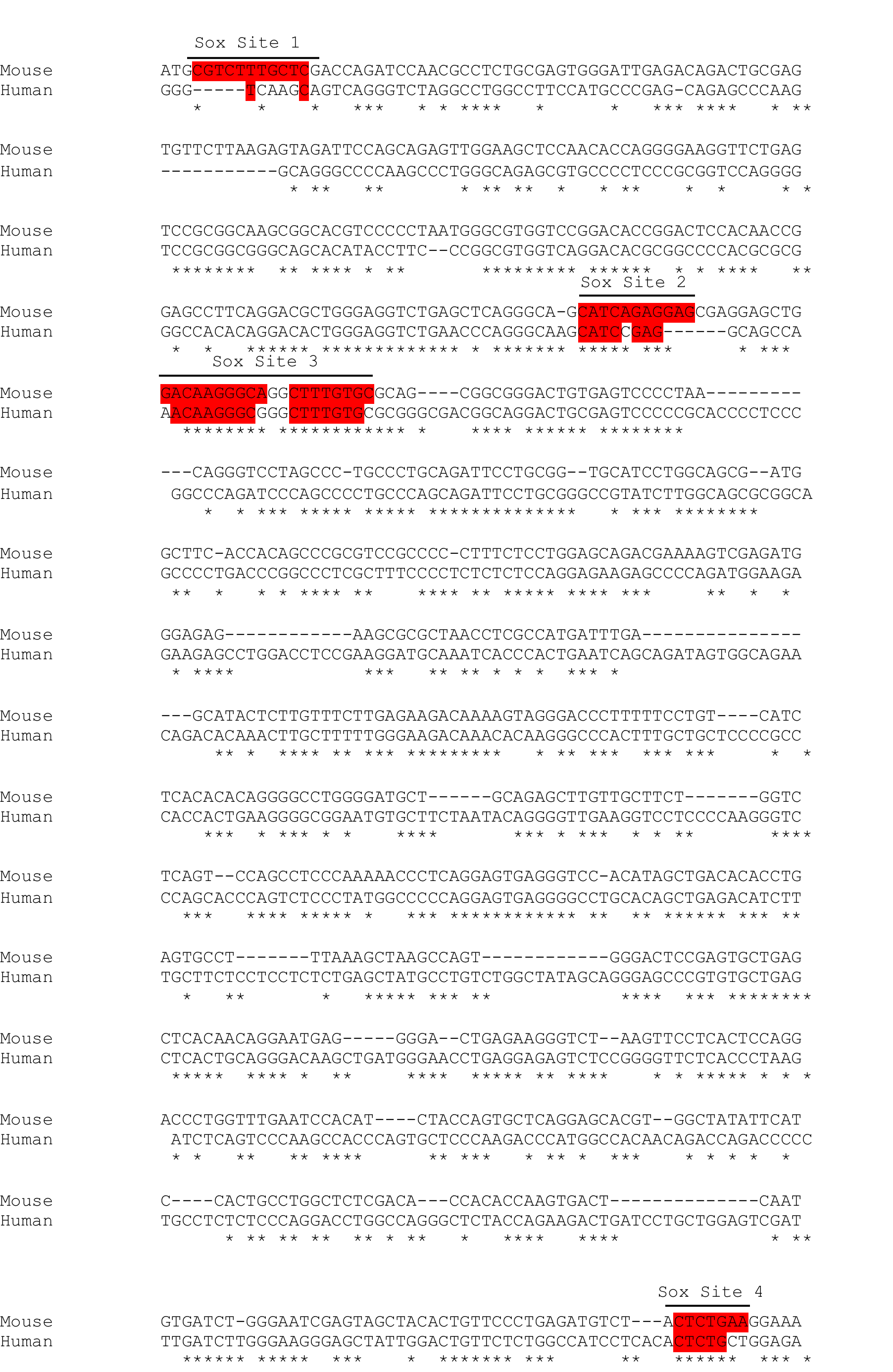

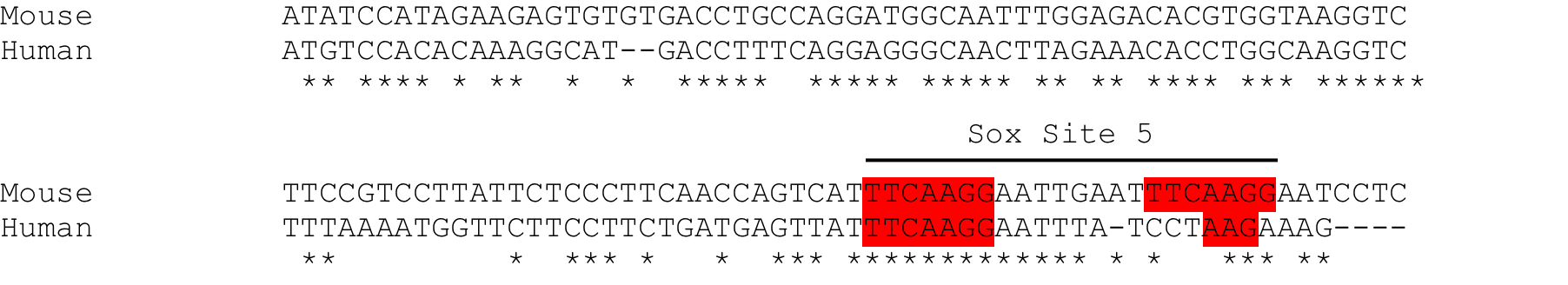


*Acan* -62

**Supplementary Figure S4:** ClustalW alignment of the -62 enhancer with possible SOX9 binding sites highlighted in red, each site is identified by the name assigned to each region. SOX9 is predicted to interact through SOX9 dimeric and monomeric interactions in the -62 *Acan* enhancer at 5 sites.


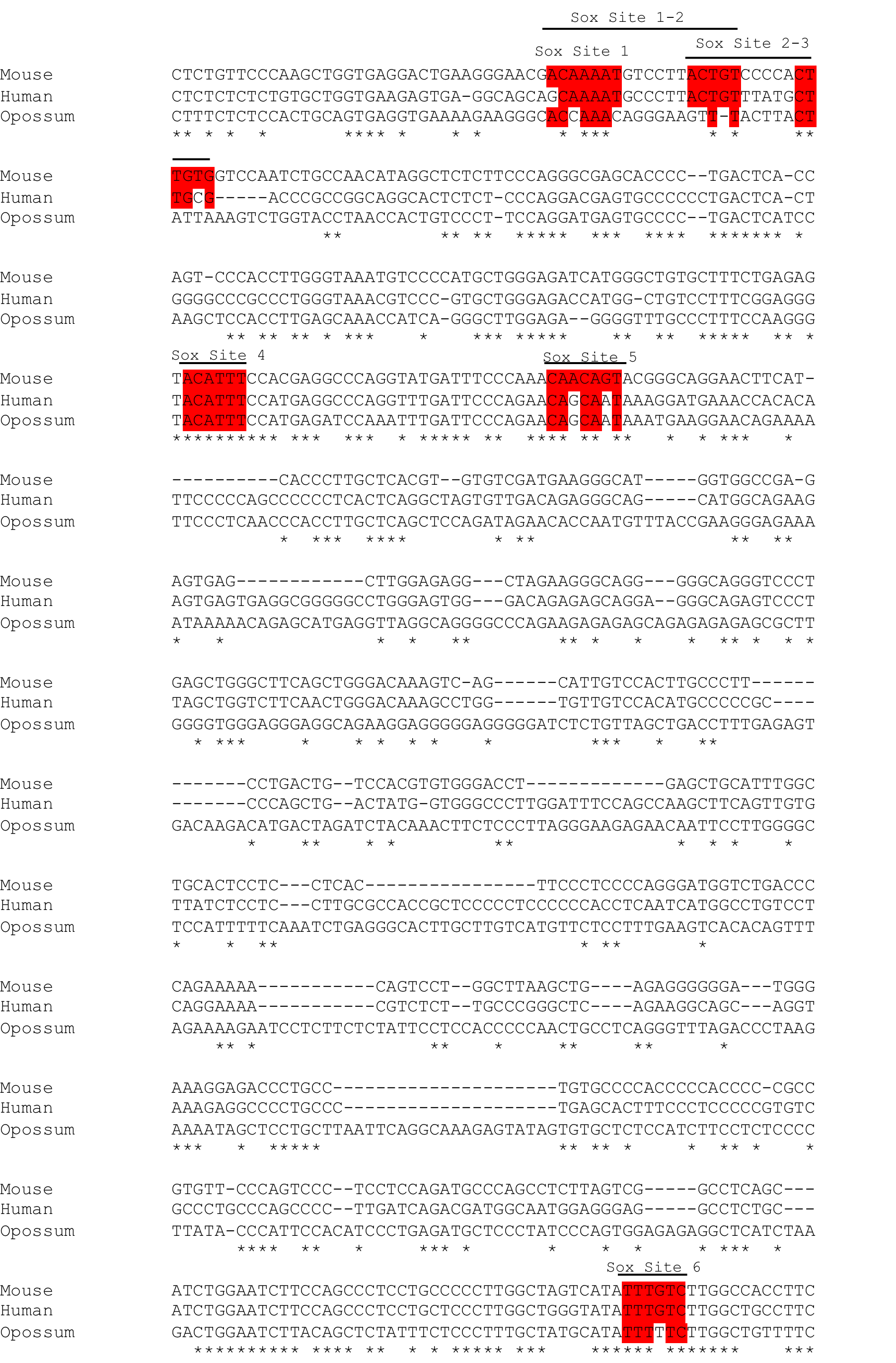

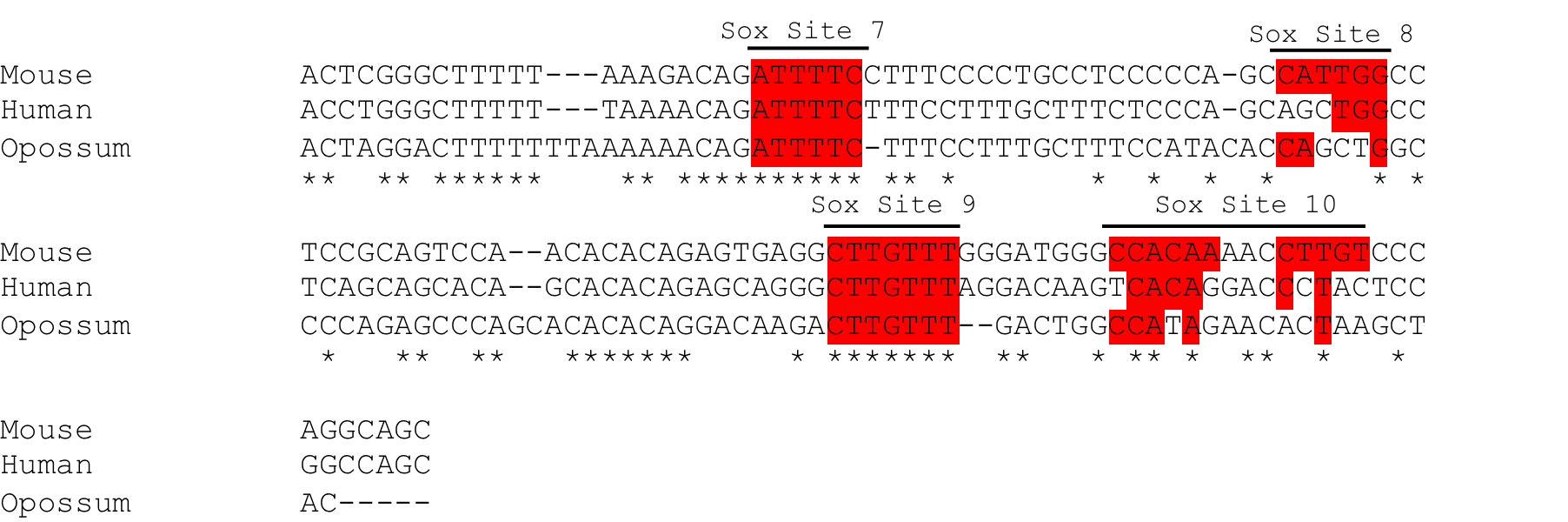


**Supplementary Figure S5:** ClustalW alignment of the -80 enhancer with possible SOX9 binding sites highlighted in red, each site is identified by the name assigned to each region. SOX9 is predicted to interact through SOX9 dimeric and monomeric interactions in the -80 *Acan* enhancer at 10 sites.

**Sequences of synthesised DNA fragments**

DNA fragments were generated by GeneArt® strings DNA fragments (Life Technologies). A single overhanging adenosine (underlined) was added to the sequence to facilitate cloning into the pCR8 TOPO vector.

Acan-30 WT

CGCATGCACACCGCCCTCCTCCACAATCCACACTGGGACTGCCTTCGGGAAGACTGTTCCCCACCTTGAACACTGCCTGCTGTCCCAACCTTCCAGCTCCTCACTCCTCCTCCTCCTCCTCACAACCTAGCTGGAAAAGGAACACAACCCTCCCTTGTGCCCTCCTGATATTTCCAGTAAAAGTACCTTGAACACAATCTTGCTGCCACAGCCCTAATTATGTGTGAAATCATTTTTTTTAAAGCTTTAAACCAAAGCAAACAAGCCCATACAGAGAGGCCCATTCTCCCAGCCTGGGAGCAGCGGGGCCTGCCTGCTGCCAGGTTACCTCAGGCTACACAGAGGCCCAGCTGTCTCACAAGCTGCCTGCCCGCCTGCCCACTGAGGTGCCTCCCCTGCCTGACTGCTGAAGA

Acan-30 Sox Mut 2_3_7

CGCATGCACACCGCCCTCCTCCACAATCCACACTGGGACTGCCTTCGGGAAGACTGTTCCCCACCTTGAACACTGCCTGCTGTCCCAACCTTCCAGCTCCTCACTCCTCCTCCTCCTCCTCACAACCTAGCTGGAAAAGGGGCTCCGCCCTCCAGGGAGCCCTCCTTGGGCCTCCCGTCACAGTACCTTGAACACAATCTTGCTGCCACAGCCCTAATTATGTGTGAAATCATTTTTTTTAAAGCTTTAAACCAAAGCAAACAAGCCCATACGGAGGGGCCCAGGCTCCCAGCCTGGGAGCAGCGGGGCCTGCCTGCTGCCAGGTTACCTCAGGCTACACAGAGGCCCAGCTGTCTCACAAGCTGCCTGCCCGCCTGCCCACTGAGGTGCCTCCCCTGCCTGACTGCTGAAGA

Acan-30 Sox Mut 2

ACGCATGCACACCGCCCTCCTCCACAATCCACACTGGGACTGCCTTCGGGAAGACTGTTCCCCACCTTGAACACTGCCTGCTGTCCCAACCTTCCAGCTCCTCACTCCTCCTCCTCCTCCTCACAACCTAGCTGGAAAAGGGGCTCCGCCCTCCAGGGAGCCCTCCTGATATTTCCAGTAAAAGTACCTTGAACACAATCTTGCTGCCACAGCCCTAATTATGTGTGAAATCATTTTTTTTAAAGCTTTAAACCAAAGCAAACAAGCCCATACAGAGAGGCCCATTCTCCCAGCCTGGGAGCAGCGGGGCCTGCCTGCTGCCAGGTTACCTCAGGCTACACAGAGGCCCAGCTGTCTCACAAGCTGCCTGCCCGCCTGCCCACTGAGGTGCCTCCCCTGCCTGACTGCTGAAGA

Acan -30 Sox Mut 3

ACGCATGCACACCGCCCTCCTCCACAATCCACACTGGGACTGCCTTCGGGAAGACTGTTCCCCACCTTGAACACTGCCTGCTGTCCCAACCTTCCAGCTCCTCACTCCTCCTCCTCCTCCTCACAACCTAGCTGGAAAAGGAACACAACCCTCCCTTGTGCCCTCCTTGGGCCTCCCGTCACAGTACCTTGAACACAATCTTGCTGCCACAGCCCTAATTATGTGTGAAATCATTTTTTTTAAAGCTTTAAACCAAAGCAAACAAGCCCATACAGAGAGGCCCATTCTCCCAGCCTGGGAGCAGCGGGGCCTGCCTGCTGCCAGGTTACCTCAGGCTACACAGAGGCCCAGCTGTCTCACAAGCTGCCTGCCCGCCTGCCCACTGAGGTGCCTCCCCTGCCTGACTGCTGAAGA

Acan -30 Sox Mut 7

ACGCATGCACACCGCCCTCCTCCACAATCCACACTGGGACTGCCTTCGGGAAGACTGTTCCCCACCTTGAACACTGCCTGCTGTCCCAACCTTCCAGCTCCTCACTCCTCCTCCTCCTCCTCACAACCTAGCTGGAAAAGGAACACAACCCTCCCTTGTGCCCTCCTGATATTTCCAGTAAAAGTACCTTGAACACAATCTTGCTGCCACAGCCCTAATTATGTGTGAAATCATTTTTTTTAAAGCTTTAAACCAAAGCAAACAAGCCCATACGGAGGGGCCCAGGCTCCCAGCCTGGGAGCAGCGGGGCCTGCCTGCTGCCAGGTTACCTCAGGCTACACAGAGGCCCAGCTGTCTCACAAGCTGCCTGCCCGCCTGCCCACTGAGGTGCCTCCCCTGCCTGACTGCTGAAGA

Acan -30 Rbpj-κ Mut

ACGCATGCACACCGCCCTCCTCCACAATCCACACTGGGACTGCCTTCGGGAAGACTGTTCCCCACCTTGAACACTGCCTGCTGTCCCAACCTTCCAGCTCCTCACTCCTCCTCCTCCTCCTCACAACCTAGCTGGAAAAGGAACACAACCCTCCCTTGTGCCCTCCTGATATTTCCAGTAAAAGTACCTTGAACACAATCTTGCTGCCACAGCCCTAATTATTTGTTAAATCATTTTTTTTAAAGCTTTAAACCAAAGCAAACAAGCCCATACAGAGAGGCCCATTCTCCCAGCCTGGGAGCAGCGGGGCCTGCCTGCTGCCAGGTTACCTCAGGCTACACAGAGGCCCAGCTGTCTCACAAGCTGCCTGCCCGCCTGCCCACTGAGGTGCCTCCCCTGCCTGACTGCTGAAGA

**Table S3:** Genomic information of *Acan* enhancer. Distance was calculated from the 3’ end of the enhancer to the transcription start site of mouse and human. Original nomenclature was taken from the 5’ end of the enhancer.

| **Enhancer** | **Mouse (mm9)** | | | **Human (hg19)** | | |
| --- | --- | --- | --- | --- | --- | --- |
|  | Coordinates | Size (bp) | Actual distance (kb) | Coordinates | Size (bp) | Actual distance (kb) |
| +28 | chr7:86,224,417-86,226,396 | 2015 | +28.0 | chr15:89,376,262-89,378,225 | 1964 | +31.6 |
| +28 Small | chr7:86,224,821-86,225,629 | 804 | +27.3 | chr15:89,376,619-89,377,482 | 864 | +30.8 |
| -30 | chr7:86,165,553-86,167,458 | 1906 | -30.9 | chr15:89,311,396-89,313,708 | 2313 | -33.0 |
| -30 Small | chr7:86,166,240-86,166,651 | 412 | -31.7 | chr15:89,312,808-89,313,168 | 361 | -33.5 |
| -62 | chr7:86,134,232-86,136,363 | 2132 | -62.0 | chr15:89,283,882-89,286,567 | 2686 | -60.1 |
| -62 Small | chr7:86,135,086-86,136,098 | 1013 | -62.3 | chr15:89,284,881-89,285,981 | 1101 | -60.7 |
| -80 | chr7:86,115,696-86,118,311 | 2616 | -80.1 | chr15:89,259,357-89,262,271 | 2915 | -84.4 |
| -80 Small | chr7:86,116,387-86,117,140 | 754 | -81.2 | chr15:89,260,172-89,260,980 | 809 | -85.7 |

**Table S4:** Summary of reporter gene expression detected in E15.5 embryos.

| **Construct** | **Expressor/ Transgenic (%)** | **Total analysed in transient transfection** |
| --- | --- | --- |
| +28 | 3/3 (100) | 16 |
| A1 | 3/5 (60) | 31 |
| -30 | 4/4 (100) | 17 |
| -80 | 8/13 (62) | 29 |
| -62 | 3/6 (50) | 20 |
| -30 Sox mut_2_3_7 | 3/3 (100) | 30 |
| -30 Sox mut_2 | 1/2 (50) | 15 |
| -30 Sox mut_3 | 4/4 (100) | 14 |
| -30 Sox mut_7 | 2/4 (50) | 9 |
| -30 rbpj_mut_C | 4/5 (80) | 20 |

**Supplementary Figure S6: Initial EMSA for the -30 enhancer SOX9 binding**


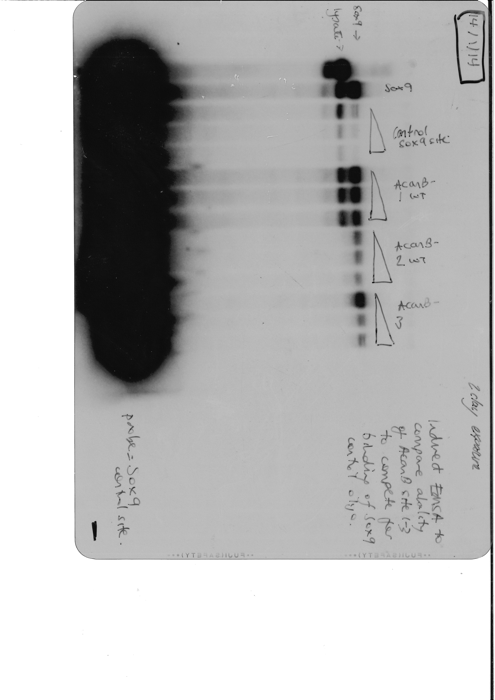

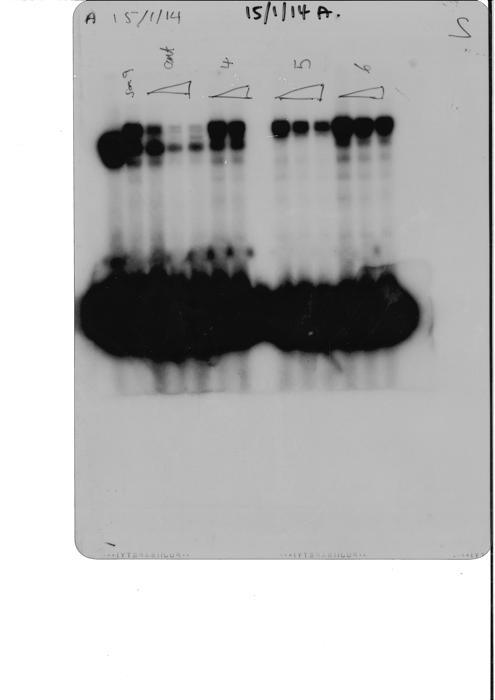

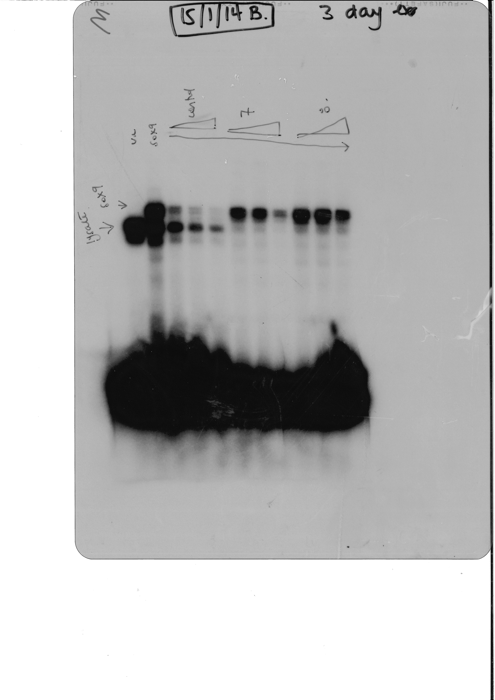

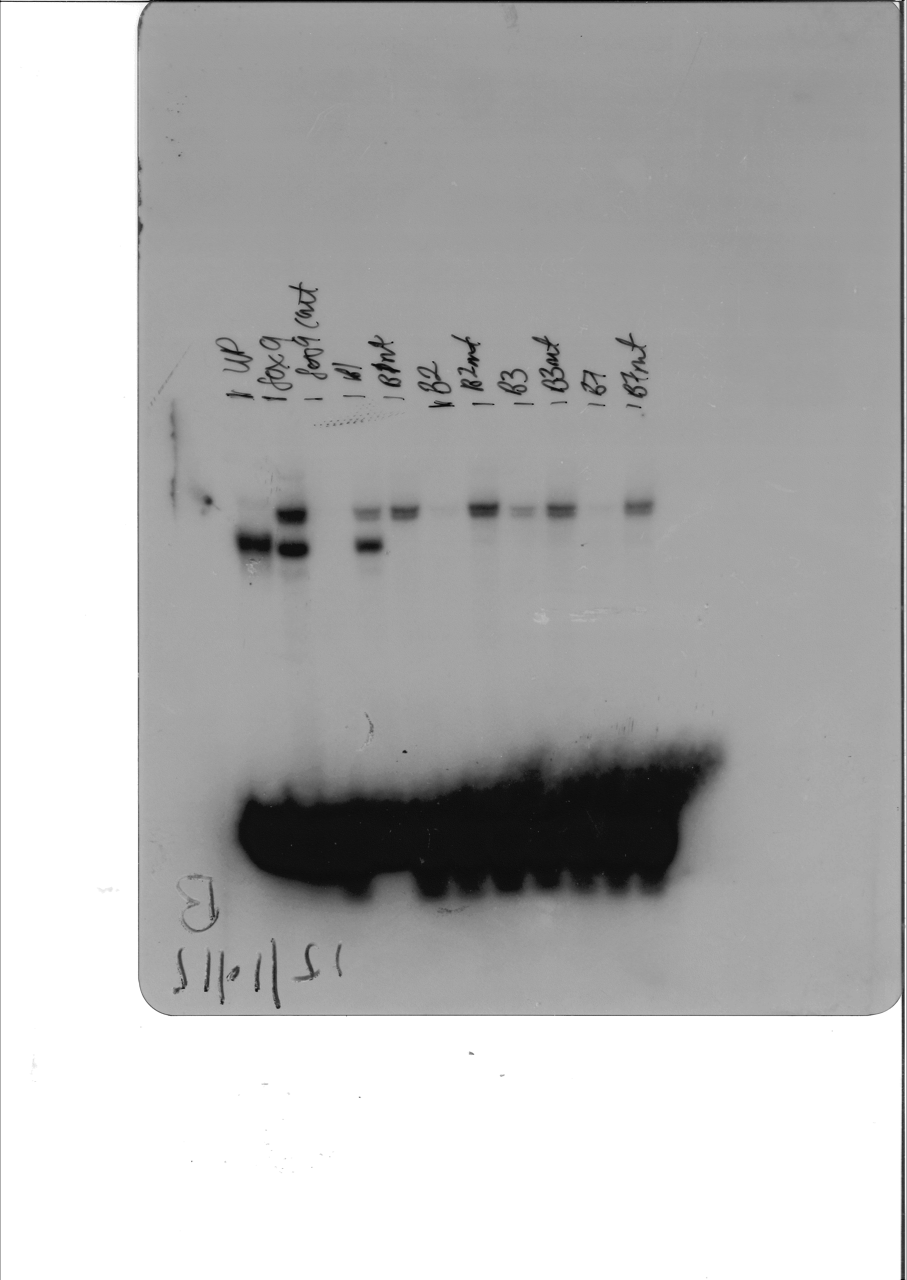


*(Top left, Top right and bottom left)* Predicted SOX9 binding site EMSAs for wild type sequences of sites 1-8 of the -30 enhancer. Initial examination competitor oligonucleotides were incubated with increasing concentration to determine if they were able to out compete for binding when compared to the *Col2a1* binding sequence. Gels were exposed on X-ray film for 48-72 hours and originals are presented here. *(Bottom right)* Original image of the -30 enhancer SOX9 sites EMSA gel from Figure 4B.

**Supplementary Figure S7: Original images for EMSA experiments**
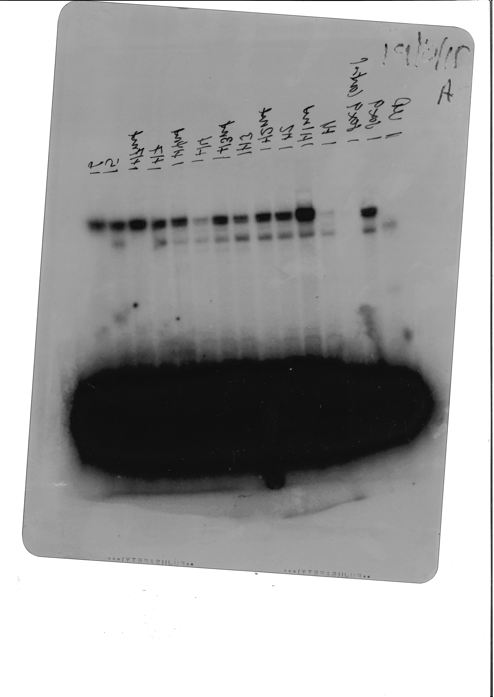

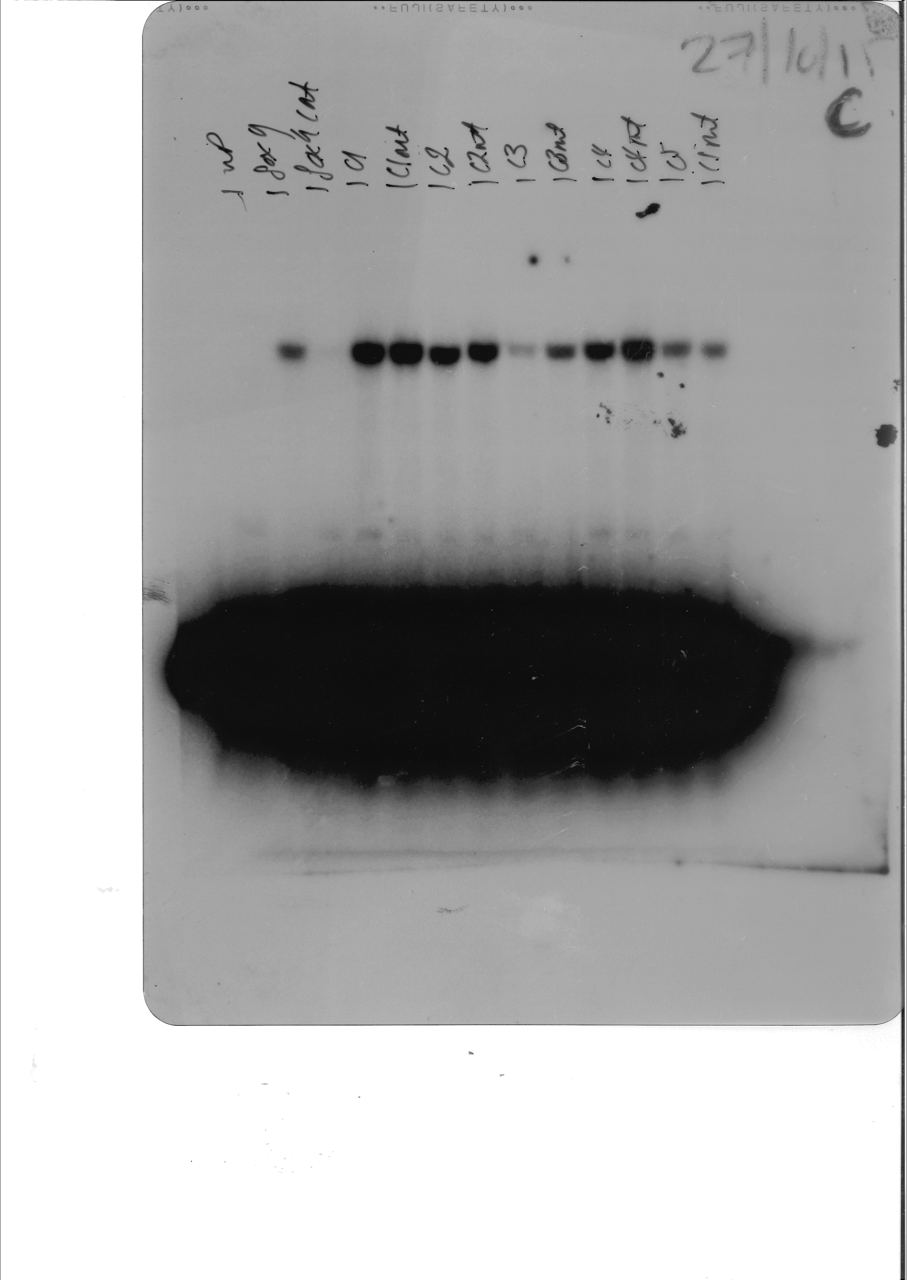

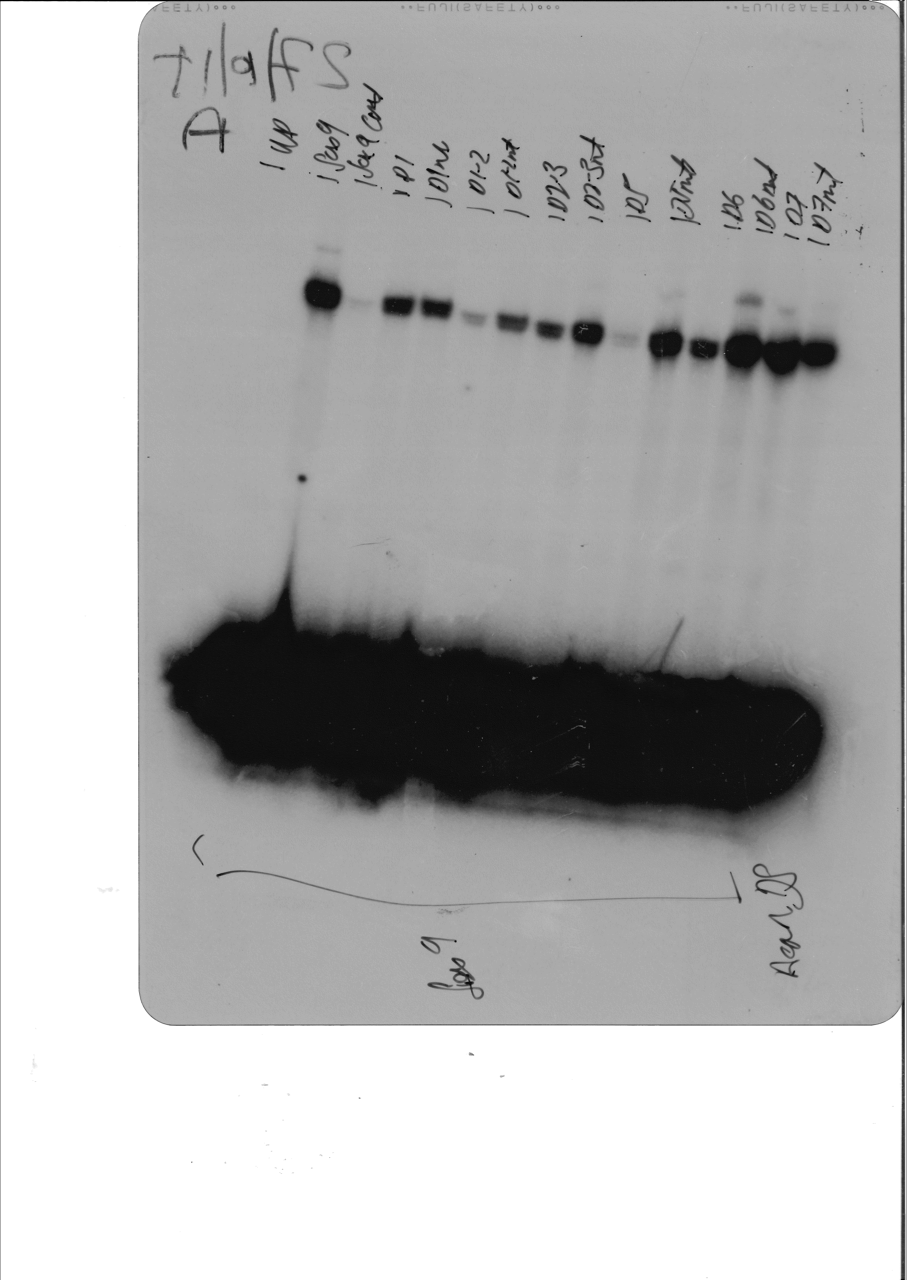

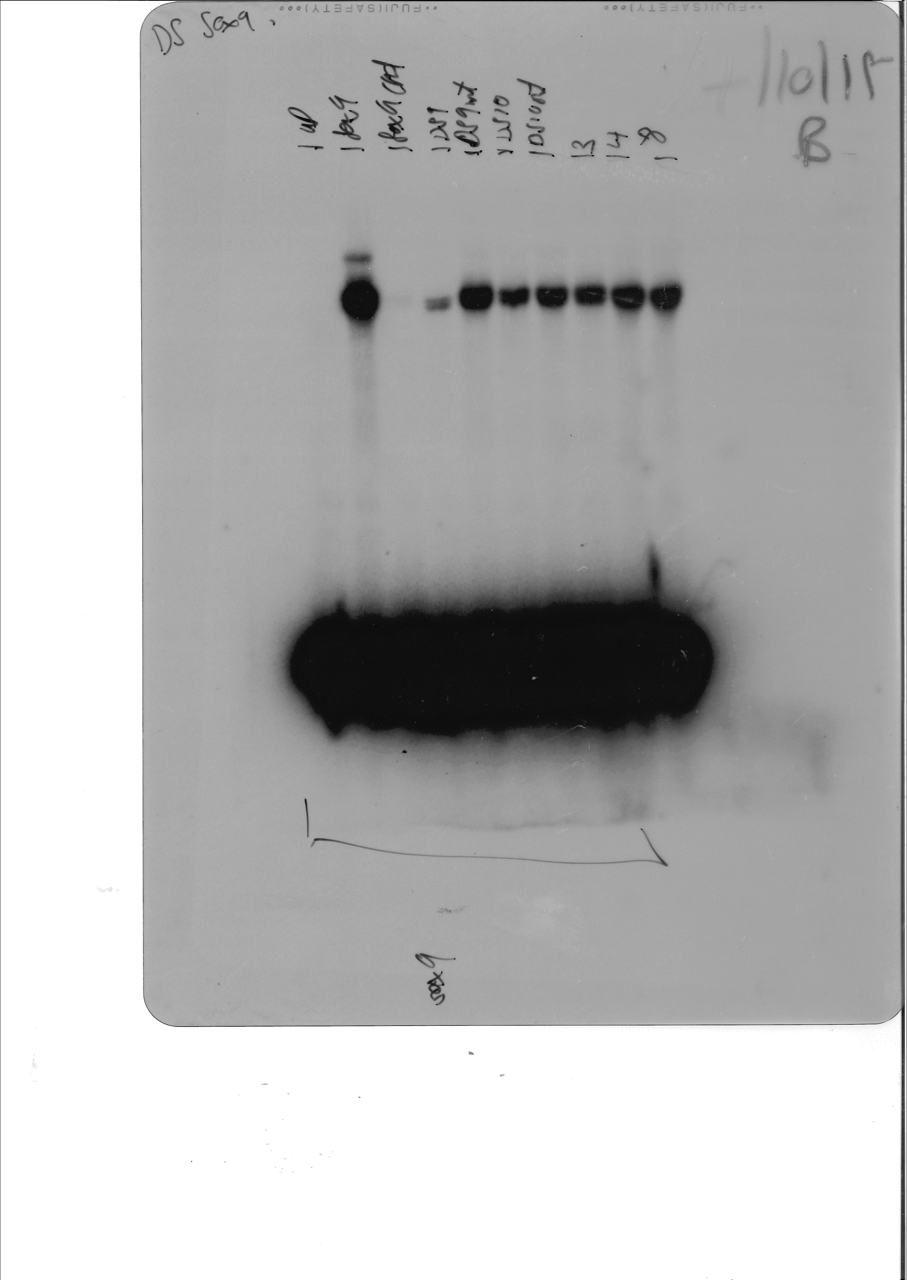


*(Top left)* Original image of the +28 enhancer SOX9 sites EMSA gel that was cropped for Figure 4C.

(Top right) Original image of the -60 enhancer SOX9 sites EMSA that was used for Figure 4D.

*(Bottom)* Original gels for the -80 enhancer SOX9 sites EMSA that were cropped for Figure 4E. Two gels were ran simultaneously and exposed for the same amount of time prior to processing.

**Supplementary Figure S8: Original image for the -30 enhancer Rbpj-κ EMSA**
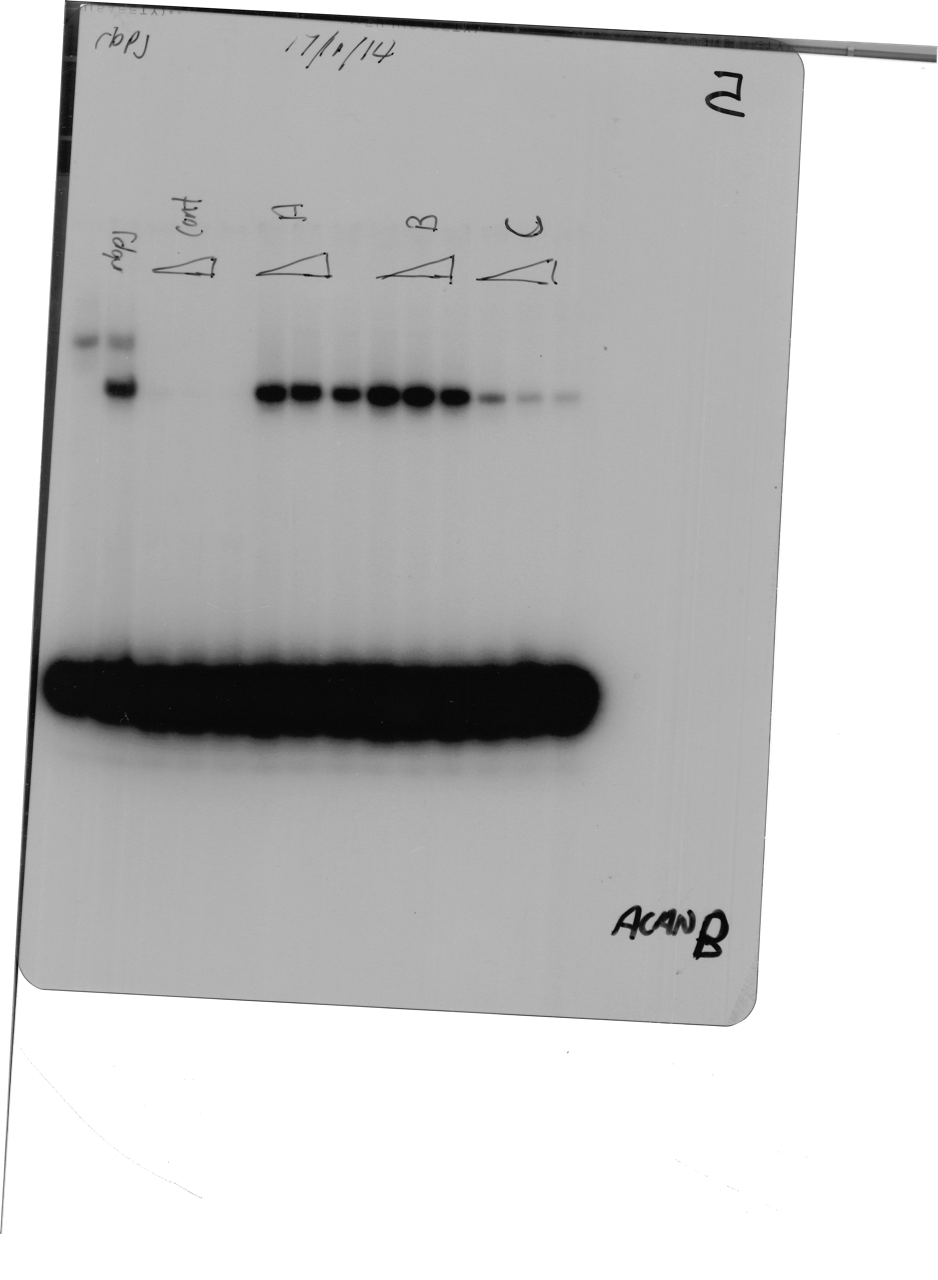


**Supplementary Figure S9:**


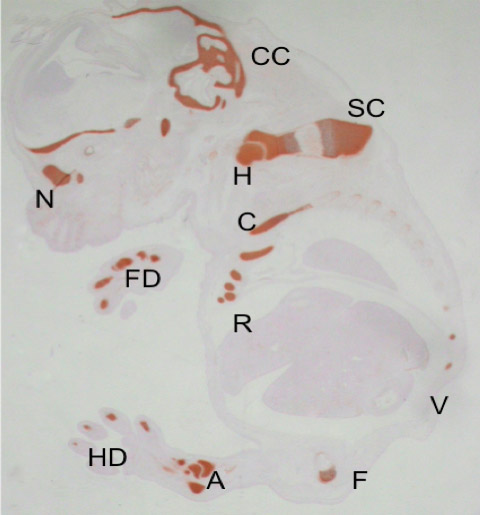

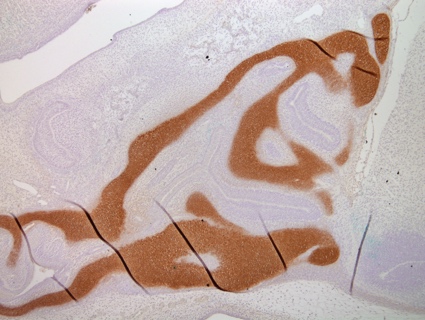

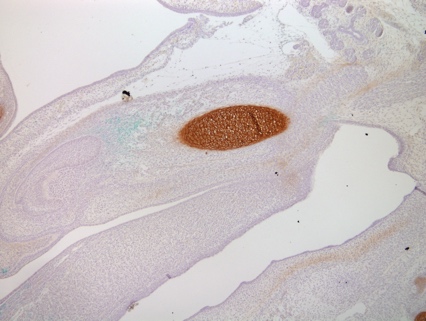

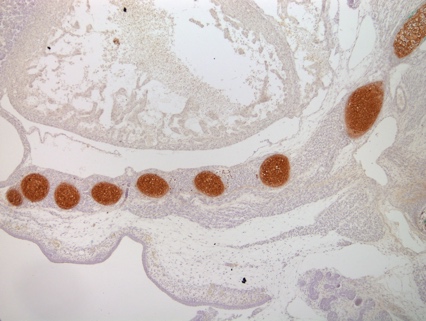

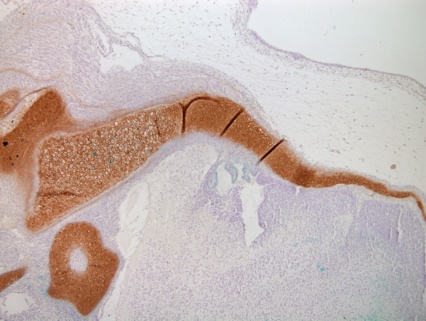


M

BO

R

E

Immunohistochemistry of Acan using BR-1 polyclonal antibody and peroxidase on sections of E15.5 embryo showing protein presence in skeletal elements of the developing embryos. A (ankle), CC (chondrocranium), E (ear cartilage), F (femur), FD (forelimb digits), H (humerus), HD (hindlimb digits), M (Meckel’s cartilage), N (nasal cartilage), RA (radius), R (rib cartilage), SC (scapular cartilage), V (vertebral cartilage), BO (basioccipital cartilage).
